# Heterodimerization of p45–p75 Modulates p75 Signaling: Structural Basis and Mechanism of Action

**DOI:** 10.1371/journal.pbio.1001918

**Published:** 2014-08-05

**Authors:** Marçal Vilar, Tsung-Chang Sung, Zhijiang Chen, Irmina García-Carpio, Eva M. Fernandez, Jiqing Xu, Roland Riek, Kuo-Fen Lee

**Affiliations:** 1 Clayton Foundation Laboratories for Peptide Biology, The Salk Institute, La Jolla, California, United States of America; 2 Neurodegeneration Unit, Chronic Disease Program, Spanish Institute of Health Carlos III, Madrid, Spain; 3 Laboratory for Physical Chemistry, ETH Zürich, Zürich, Switzerland; McGill University, Canada

## Abstract

The p75 neurotrophin receptor, a member of the tumor necrosis factor receptor superfamily, is required as a co-receptor for the Nogo receptor (NgR) to mediate the activity of myelin-associated inhibitors such as Nogo, MAG, and OMgp. p45/NRH2/PLAIDD is a p75 homologue and contains a death domain (DD). Here we report that p45 markedly interferes with the function of p75 as a co-receptor for NgR. P45 forms heterodimers with p75 and thereby blocks RhoA activation and inhibition of neurite outgrowth induced by myelin-associated inhibitors. p45 binds p75 through both its transmembrane (TM) domain and DD. To understand the underlying mechanisms, we have determined the three-dimensional NMR solution structure of the intracellular domain of p45 and characterized its interaction with p75. We have identified the residues involved in such interaction by NMR and co-immunoprecipitation. The DD of p45 binds the DD of p75 by electrostatic interactions. In addition, previous reports suggested that Cys257 in the p75 TM domain is required for signaling. We found that the interaction of the cysteine 58 of p45 with the cysteine 257 of p75 within the TM domain is necessary for p45–p75 heterodimerization. These results suggest a mechanism involving both the TM domain and the DD of p45 to regulate p75-mediated signaling.

## Abstract 2

The formation of a p45-p75 heterodimer overrides p75â€™s inhibition of nerve regeneration by stopping p75 homodimers from forming and creating a complex with the Nogo receptor.

## Author Summary

Injuries to the brain and spinal cord often result in paralysis due to the fact that the injured nerves cannot regrow to reach their normal targets and carry out their functions. At the injury sites, there are proteins released from the damaged myelin that bind the Nogo receptor (NgR) on the nerve and inhibit its regeneration. The NgR needs to form a complex with the p75 neurotrophin receptor in order to mediate this inhibitory signal. Here we found that p45, a homologue of p75, can also bind to p75 and block its inhibitory activity when overexpressed. To perform its function, p75 needs to dimerize through both its transmembrane and intracellular domains, facilitating the recruitment of several proteins. Our structural and functional studies show that p45 binds specifically to conserved regions in the p75 transmembrane and the intracellular domain and that this blocks p75 dimerization along with its downstream signaling. Thus, this study demonstrates that altering the oligomerization of p75 is a good strategy to override p75's inhibitory effects on nerve regeneration, and it opens the door for the design of specific p75 inhibitors for therapeutic applications.

## Introduction

The neurotrophin receptor p75 is a member of the tumor necrosis factor receptor (TNFR) superfamily and has four extracellular cysteine rich domains, a single transmembrane (TM) domain, and an intracellular domain (ICD) comprising a juxtamembrane and a death domain (DD) [1]–[5]. Depending on co-receptor partners and cellular contexts, p75 may play seemingly opposing effects in multiple systems. For example, p75 interacts with Trk receptors to promote neurotrophin-dependent nerve growth. In contrast, p75 has been shown to play a role in apoptosis when binding to pro-neurotrophins and with the co-receptor sortilin [4]. In addition, p75 inhibits nerve growth mediated by myelin-associated inhibitors via functioning in part as a co-receptor for the GPI-linked neuronal Nogo-66 receptor (NgR) [6] or another non-NgR molecule that is yet to be identified [7],[8]. Elucidation of the mechanisms that modulate p75-mediated signaling may increase our understanding of neural development and nerve injury.

Upon nerve injury in adult mammals, factors at the injury site such as myelin-associated inhibitors inhibit regeneration of injured axons, resulting in permanent disability. Axon regeneration is blocked by the presence of multiple types of nerve growth inhibitors, such as myelin-associated inhibitors from damaged myelins, chondroitin sulphate proteoglycans, and repulsive axon-guidance molecules expressed by reactive glial cells [9]–[12]. The structurally dissimilar myelin-associated inhibitors Nogo66, MAG, and OMgp inhibit axon growth by binding to the NgR, a GPI-linked protein, which then transduces the inhibitory signal into the cell by binding to co-receptors with intracellular signaling domains, such as p75 [13],[14] or TROY [15],[16]. LINGO-1 also plays a role in NgR signaling [17]. Downstream from their receptor binding, these myelin inhibitors trigger inhibition of axonal growth through the activation of the small GTPase Rho [18]–[21] in a protein kinase C (PKC)-dependent manner [22]. Targeting this complex has been described to lead to the promotion of neurite outgrowth, oligodendrocyte proliferation and differentiation, and inhibition of cell death.

p45 is highly homologous in sequence to p75. It is also called neurotrophin receptor homologue 2 (NRH2) [25], neurotrophin receptor alike DD protein (NRADD) [24], or p75-like apoptosis inducing DD protein (PLAIDD) [23]. P45 displays strong sequence similarity to p75 in the TM, juxtamembrane, and DD regions [26]. P45 contains a truncated and short extracellular domain (ECD) with no neurotrophin binding domain.

It has been shown previously that p45 associates with p75 and with TrkA receptors [23],[25],[27]. In addition, p45 participates in the trafficking of sortilin to the plasma membrane [27]. However, its role in other p75-regulated signaling pathways has not been studied. In this study, we have explored the modulation of p75/NgR signaling. The results indicate that p45 heterodimerizes with p75 and, thereby, impedes the formation of p75 homodimer that is required for the p75/NgR complex formation and its downstream activation of RhoA GTPase. In addition, we found that p45 binds p75 through both the TM and the ICDs. Furthermore, we showed that a cysteine–cysteine interaction within the TM domain of p45 and p75 is required for stabilization of their heterodimer formation. The results reveal a new mechanism of modulating p75-mediated inhibitory signaling via heterodimer formation with a member of the TNFR superfamily such as p45.

## Results

### p45 Forms a Stable Complex with p75 and Is Up-Regulated upon Nerve Injury

**Figure 1:**
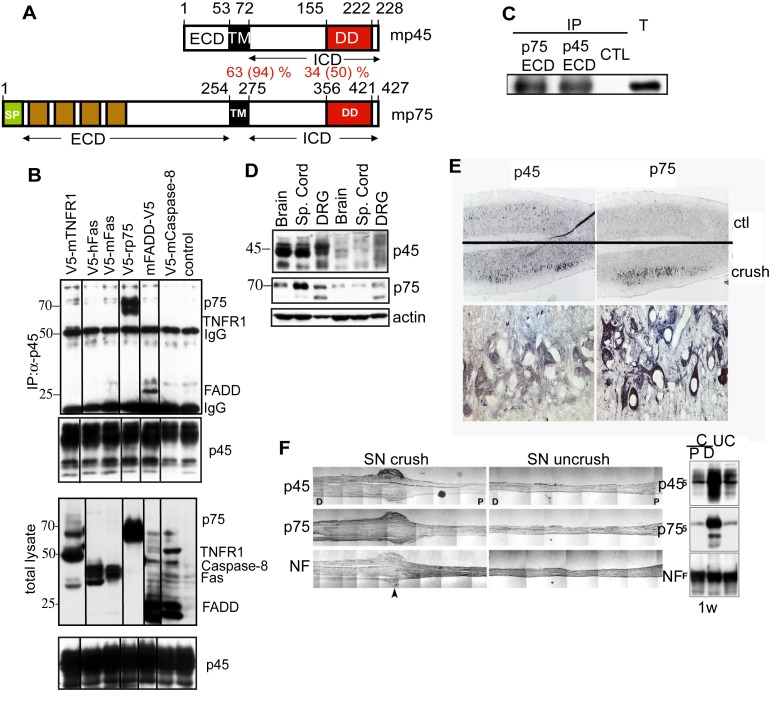
Interactions between p75 and p45. (A) A schematic diagram showing domains of p45 in comparison with p75. TM, transmembrane domain; DD, putative death domain; PDZ, putative PDZ binding domain. The degrees of identity and homology in amino acid residues between mouse p75 and p45 are shown in percentages. (B) P45 forms a complex with FADD and p75. V5-tagged TNFR1, human Fas, mouse Fas, p75, FADD, or Caspase-8 was transfected into CrmA/Flag-p45/293 stable cells. The lysates were immunoprecipitated with anti-Flag antibodies and immunoblotted with anti-V5 antibodies. (C) P7 cerebellum extracts were immunprecipitated with anti-p75 ECD antibody (9651) or anti-p45 ECD antibody (6750) followed by immnuoblotting with anti-p75 antibody (Buster). T, total lysate. (D) Western blotting analysis of p45 and p75 expression in the brain, spinal cord, and DRG of P0 and adult mice. (E) Increased expression of p45 and p75 in the spinal cord and sciatic nerve following sciatic nerve injury. (Top) Spinal cord sections were immunostained with anti-p45 or -p75 antibodies. p45 and p75 immunoreactivities were markedly increased in the ipsilateral side as compared to the contralateral side. Higher magnification indicated that expression of p45 and p75 is increased in motor neurons. (F) Longitudinal sections of crushed and uncrushed sciatic nerves were immunostained with antibodies against p45, p75, or neurofilament. The levels of p45 and p75 were markedly increased in the distal (D) portion of sciatic nerves as compared to the proximal (P) end and the uncrushed (UC) nerve.

p45 contains a DD (Figure 1A). Because the function of DDs is to bind the DD of other members of the DD superfamily in order to transduce signals [28], we investigated whether p45 can interact with other members of the DD superfamily, such as p75, FADD, TNFR1, and Fas. As shown in Figure 1B, p45 can be co-immunoprecipitated with p75 or FADD when co-expressed in 293 cells, but not with caspase-8, Fas, or TNFR1. Recently we characterized the interaction of p45 with FADD and its role upon spinal cord injury [29]. In the present study, we focus on the interaction of p45 with p75.

p45 and p75 share a high degree of amino acid similarity in their TM domain (94%), including conserved cysteine residues [30], and the ICD (50%) (Figure 1A). However, the ECD of p45 is short and has no binding sites for neurotrophins (Figure 1A). p45 and p75 are expressed in both the peripheral nervous system (PNS) and central nervous system (CNS) during development [26]. We found that p75 and p45 form an immunocomplex in cerebellum extracts (Figure 1C). The expression level of p45 is high in embryonic but is significantly reduced in adult tissues (Figure 1D). However, p45 is up-regulated after sciatic nerve injury in the spinal cord and sciatic nerve in a similar fashion as p75 (Figure 1E,F). Because p45 shares a similar protein sequence with p75 and has similar up-regulated expression patterns with p75 after injury, we decided to investigate whether p45 regulates signaling mechanisms involving p75.

### p45 Interferes with p75/NgR Signaling

**Figure 2:**
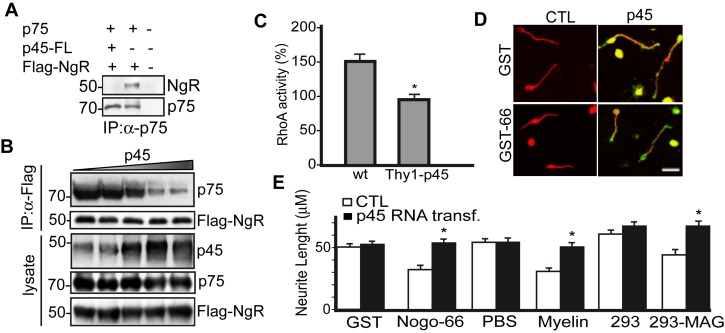
p45 interferes with the p75-NgR interaction and signaling. (A) Lysates from HEK293 cells co-transfected with vectors expressing p45, p75, and Flag-tagged human NogoR (Flag-hNgR) were immunoprecipitated with anti-p75 antibodies and probed with anti-Flag antibodies. The results showed that p75 and hNgR form a complex and presence of p45 reduces the formation of p75/hNgR complex. (B) Co-transfection with varying amount of p45-expressing vectors markedly reduced p75/hNgR complex formation in a concentration-dependent fashion. (C) Quantification of the increase of RhoA activity after MAG-Fc treatment. In wild-type (WT) CGN cultures, when treated with MAG-Fc, the RhoA activity increased by 50%. However, in CGN cultures derived from Thy1-p45 transgenic mice, the increase in RhoA activity induced by MAG-Fc treatment is completely abolished. The value is expressed as percent over control. The value is derived from three independent experiments. * *p*<0.05, Student's *t* test. The data can be found in Table S1. (D) CGNs were seeded on glass coverslips coated with inhibitory substrates, grown for 14–18 h, and immunofluorescently stained with Tuj1 (Red) and anti-p45 (Green) antibodies. Scale bar, 50 µm. (E) Quantitative analysis of neurite length from the outgrowth assay, using Nogo66-GST, myelin, or HEK293 cells expressing MAG as inhibitory substrates. The data are represented as mean ± SEM (* *p*<0.001) and can be found in Table S1. Overexpression of p45 promoted neurite outgrowth of CGNs on inhibitory substrates.

p75 mediates nerve growth inhibition by myelin-associated inhibitors via functioning in part as a co-receptor for GPI-linked neuronal NgR [6]. Because p45 and p75 form a stable complex (Figure 1B), we investigated whether p45 interferes with or enhances the formation of the complex between p75 and NgR and subsequent signaling through the p75/NgR complex. When HEK293 cells were co-transfected with p75 and Flag-tagged human NgR (Flag-hNgR) expression constructs followed by immunoprecipitation with anti-p75ICD antibodies, we found that p45 markedly reduced the levels of complex formation between p75 and NgR (Figure 2A) and it does in a concentration-dependent manner (Figure 2B). As a control, p45 is not able to bind to NgR directly (Figure S1). To further characterize the domains of p45 involved in such inhibition, we made several deletion constructs of p45 (Figure S2 and Table S1). As shown in Figure S2, both p45 intracellular and TM domains are necessary for p45 inhibition of p75/NgR complex formation.

We examined the possibility that p45 would antagonize signaling through the p75/NgR complex. Previous results demonstrated that p75 is required for MAG-induced RhoA activation through NgR [20],[31]. RhoA activation is necessary for neurite outgrowth inhibition mediated by myelin-associated inhibitors. Thus, we examined whether overexpression of p45 blunts signaling through the p75/NgR complex. Postnatal day 7 (P7) cerebellar granule neurons (CGNs) that express low levels of endogenous p45 were transfected with full-length, capped p45 RNAs containing poly(A) tails generated by an *in vitro* transcription system. As shown in Figure S3A, the level of p45 protein was markedly elevated 24 h following RNA transfection. The cultures were then serum-starved and treated with Fc or MAG-Fc proteins. The level of activated RhoA was measured. As illustrated in Figure S3B, overexpression of p45 blocks MAG-Fc–induced RhoA activation. We also quantitatively measured RhoA activation using the G-LISA kit (Cytoskeleton Inc.) on CGN cultures from wild-type mice and Thy1-p45 transgenic mice that consistently overexpress p45 under a Thy1 promoter [29]. As shown in Figure 2C, MAG-Fc treatment of the WT CGNs induced 50% increase in the RhoA activity, whereas the MAG-Fc–induced RhoA activation is completely abolished in Thy1-p45 CGNs (Table S1). These results suggest that p45 is capable of effectively blocking RhoA activation through the p75/NgR complex. We then examined whether overexpression of p45 prevents neurite outgrowth inhibition induced by Nogo66, MAG, or CNS myelin. P7 CGNs were transfected with p45 RNAs, plated onto dishes previously coated with different substrates, and allowed to grow overnight. The cultures were double immunostained with antibodies against p45 (green) and neurotubulin (TuJ1, red) (Figure 2D). The neurite length of control CGNs and transfected CGNs that display increased p45-immunoreactivity over control CGNs was measured. As shown in Figure 2E, neurite outgrowth inhibition elicited by Nogo66 is alleviated by p45 overexpression (Table S1). Similarly, p45 overexpression significantly promotes neurite outgrowth that was otherwise inhibited when cultured on dishes coated with CNS myelin or MAG-expressing cells (Figure 2E). These results support the idea that p45 promotes neurite outgrowth.

It is worth noting that despite NgR has been implicated in mediating nerve growth inhibition induced by myelin inhibitors in culture, neurite outgrowth of CGNs from NgR null mutants is still inhibited by myelin inhibitors [7],[8]. Recent results suggest that NgR is required only for the acute growth cone-collapsing but not chronic growth-inhibitory actions of myelin inhibitors [32]. Furthermore, no measurable corticospinal tract regeneration was observed in mice lacking all Nogo isoforms [33]–[35] (but see Cafferty et al. [36]). In contrast, inhibition of nerve growth by myelin inhibitors is significantly reduced in p75-deficient CGNs [7],[8]. These results raise the possibility that a yet to be identified receptor mediates myelin inhibitor activity through p75.

### Dimerization of p75DD in Solution

**Figure 3:**
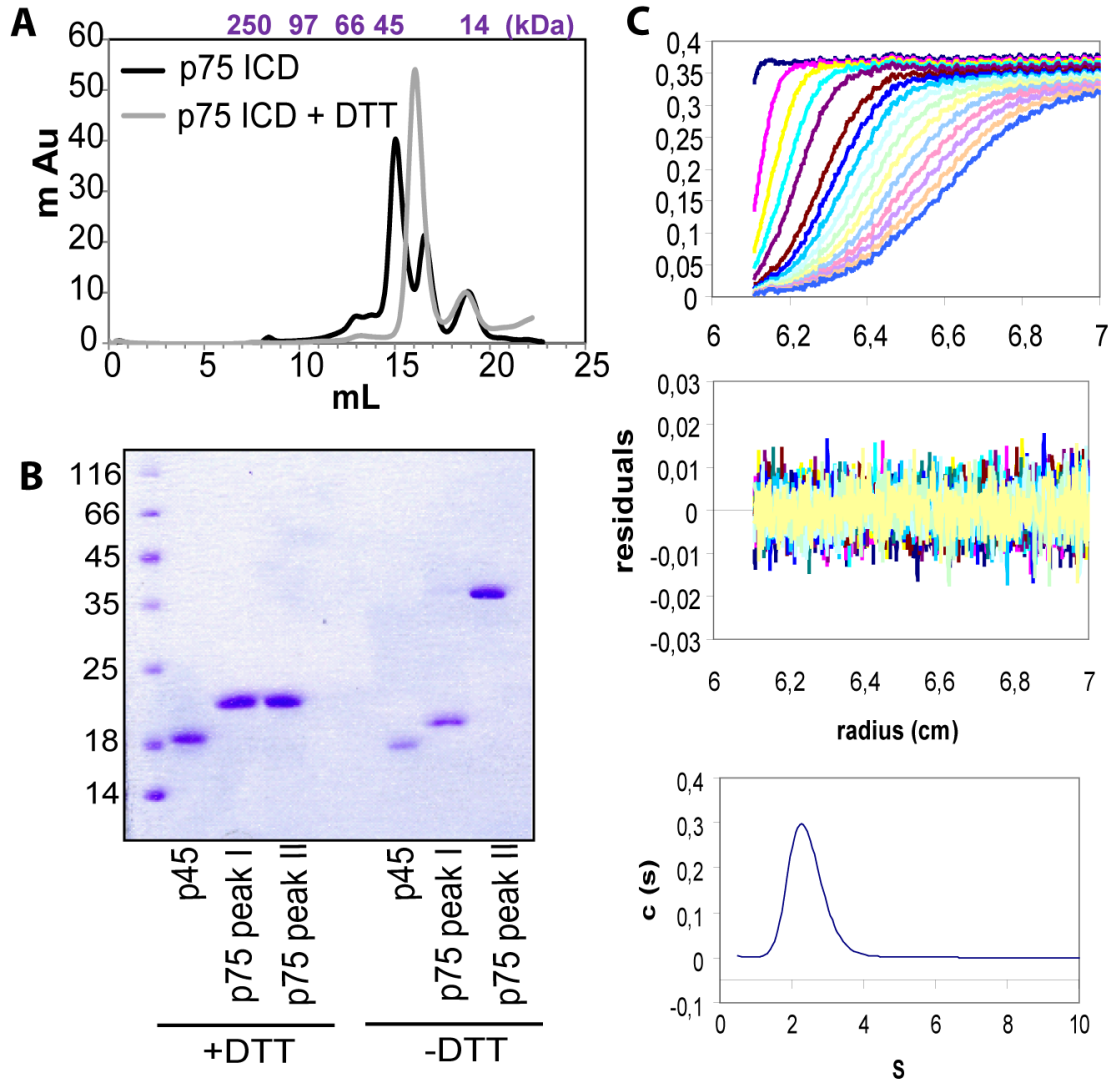
p75ICD homodimerization. (**A**) Size exclusion gel-filtration chromatography of p75ICD. The elution profile reveals the presence of a mixture of monomers and dimers in the case of p75ICD (black lines). In the presence of DTT, only one elution peak in the gel filtration chromatogram is seen (gray line). Molecular weight standards are shown above the chromatogram. (B) Coomassie blue staining of reducing and nonreducing SDS-PAGE of the fractions collected in gel filtration as shown in (A). The presence of a protein band corresponding to a p75ICD dimer in the nonreducing SDS-PAGE is shown with an arrowhead. The migration of p45ICD is shown as a reference. (C) Analytical ultracentrifugation data on p75ICD in PBS (pH 8.0). (Top) Overlay of successive sedimentation velocity profiles recorded at ∼10 min intervals, represented by different colors. The solid lines represent the direct fitting of the data to a two-species model by the Svedberg program. (Bottom) Sedimentation velocity AUC profiles and the *c(S*) distributions for p75ICD (at 0.1, 0.3, and 1.0 mg ml^−1^). The residual differences between the experimental data and the fit for each point are shown above. Theoretical p75ICD MW  = 16.5 kDa. Fitting data MW  = 30.7±1.2 kDa.

To understand the mechanism by which the p75–p45 interaction regulates p75-dependent signaling, we first characterized biophysically the ICD domain of p75. Because the DD of p75 is a protein–protein interaction motif and often is involved in functionally essential homo- and hetero-associations [37]–[40], we studied the oligomerization behavior of p75ICD comprising residues 290–418. We used gel filtration chromatography of purified p75ICD to analyze the oligomerization state of p75ICD in phosphate buffer at pH 8.0. Purification of p75ICD from bacteria yielded two peaks in the elution profile of gel filtration (Figure 3A, black lines) with the presence of some high molecular weight aggregates (Figure 3A, asterisk). After running SDS-PAGE of the peak fractions in reducing and nonreducing conditions, we found that the elution fraction I corresponds to a covalently disulfide bond dimer and fraction II to a monomer molecular weight (Figure 3B). When we purified p75ICD with the presence of DTT, a single peak was observed that eluted between the monomer and dimer peaks (Figure 3A). The same behavior is observed in the presence of iodoacetamide, which blocks free cysteines, in the lysis buffer (Figure S4). The estimated molecular weight of this fraction from the gel filtration yields a mass of 35 kDa (p75ICD MW is 16 kDa), suggesting the presence of a dimer. We carried out an analytical ultracentrifugation analysis of purified p75ICD in the presence of DTT, using equilibrium sedimentation and velocity experiments of recombinant p75ICD in the same buffer (Figure 3C). Our ultracentrifugation confirms that p75ICD behaves in solution as a single species with a molecular weight of 30.7 kDa, close to the theoretical dimer of p75ICD (see Figure 3C legend). Altogether, we conclude that p75ICD is a noncovalent dimer that during purification or in oxidative conditions dimerizes through a disulfide bond (Figure S5). Recently the crystal structure of a covalent disulfide p75ICD dimer, purified from bacteria in oxidative conditions (in the presence of DTNB), has been described [41]. The dimer is mediated by a covalent disulfide bond through Cys379. We think that the dimer found in this work is the same dimer, because when we made p75-C379A mutant, gel filtration gives a monomer peak (Figure S6). Whether this covalent disulfide dimer is formed in the reducing conditions encountered inside cells and what its biological function is need to be further investigated.

### p75ICD Dimer Interface Mapping by NMR

**Figure 4:**
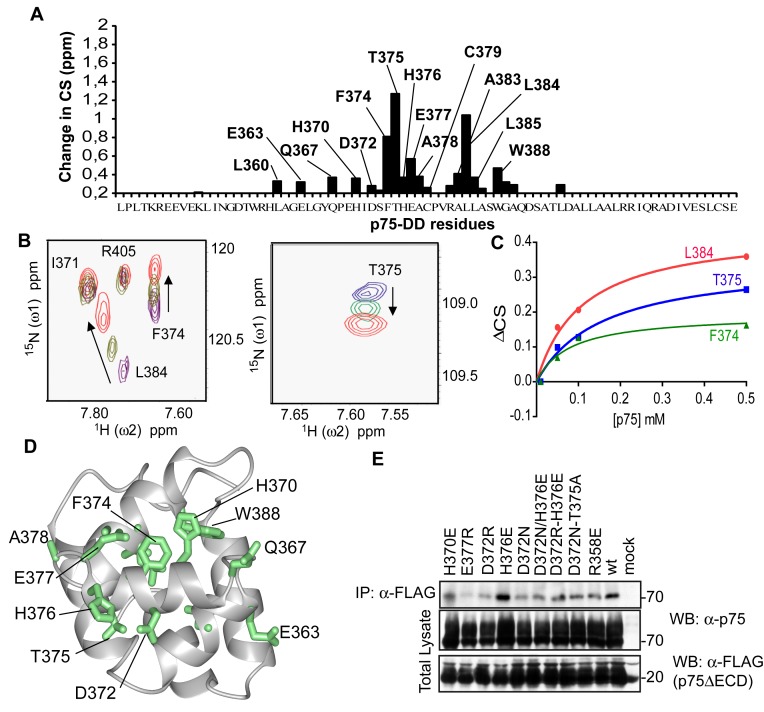
Determination of p75ICD dimer interface by NMR. (A) Concentration-dependent chemical shift changes of ^15^N-labeled p75ICD observed in [42]-TROSY spectra at p75ICD concentrations of 10 µM, 100 µM, and 500 µM. (B) Some examples of residue peaks of p75ICD at the different concentrations of 10 µM (purple), 100 µM (green), and 500 µM (red). The cross-peaks are labeled to the corresponding residues. (C) Determination of monomer-dimer k_d_ using the changes in the chemical shift of different residues from the dimer interface plotted versus p75ICD concentration. (D) Concentration-dependent chemical shift changes from data in (B) are mapped onto the 3D structure of p75DD (PDB code 1NGR; [45]). The 3D structure is represented by a ribbon diagram and by a surface representation. Residues with chemical shift perturbations ΔCS, larger than 0.2 ppm, are displayed and colored in green. ΔCS  = 25[Δ(δ(^1^H))^2^ + Δ(δ(^15^N))^2^]^0.5^, where δ(^1^H) and δ(^15^N) are the chemical shifts in part per million (ppm) along the ω_2_(^1^H) and ω_1_(^15^N) dimensions, respectively. (E) Immunoprecipitation experiments of wild-type or some p75 mutants in the dimer interface demonstrating the role of those residues in p75 homodimerization.

NMR titration experiments were performed at different p75ICD concentrations in the presence of DTT to avoid formation of the disulfide dimer. Concentration-dependent chemical shift changes in [42] TROSY spectra [43] of ^15^N-labeled p75ICD were then used to map the homodimer interface (Figure 4A). Figure 4B shows several examples of concentration-induced chemical shift changes. The appearance of only one peak suggests a slow monomer–dimer equilibrium for NMR time scale measurements. This pattern of change is indicative of a weak binding that we can estimate from plotting changes in the chemical shift of interface residues. We obtained a K_d_ of ∼100 µM (Figure 4C). Such low binding affinities are typically observed for DD-type interactions [44]. Residues with the most pronounced chemical shift changes—that is, L360, E363, Q367, H370, D372, F374, T375, C379, H376, E377, A383, L384, L385, and W388, (Figure 4A)—were then mapped onto the reported NMR structure of p75ICD (Figure 4D) [45]. These residues are all located on one side of the DD, in particular within helices α3 and α4. Together with the presence of only one TROSY cross-peak per ^15^N-^1^H moiety, these results suggest the formation of a symmetrical p75-DD homodimer. Because helices α3 and α4 contain charged amino acid residues, the homodimer formation may be in part due to electrostatic interactions. Recently the crystal structure of an asymmetrical dimer of p75-DD has been described [41]. In that structure, R404 from monomer A interacts with S373, H376, and E377 of monomer B. In our NMR titration study, chemical shift changes of R404 are not observed (Figure 4A), although changes in the chemical shift of S373 (small) and E377 and H376 (big) are clearly visible (Figure 4A). These results suggest that in solution the symmetrical dimer is favored, although we cannot exclude the possibility that p75ICD with different conformations is present in solution. Sometimes crystallization favors conformations that are better packed but are not necessarily the prevalent conformations in solution.

To further characterize and confirm the homodimer interface in the full-length p75, we made p75 mutant constructs containing amino acid replacements at different residues, which showed high concentration-dependent chemical shift changes in the NMR studies of p75ICD (Figure 4A). The emphasis of the amino acid replacements was charge changes due to the potential electrostatic nature of the interaction (D372R, H376E, and E377R; Figure 4E). Wild-type or mutant p75 constructs were co-transfected with a Flag-tagged construct that contained only the TM domain and the ICD of p75 (Flag-ΔECD-p75) in HEK293 cells. The presence of the p75 dimers was measured by co-immunoprecipitation with an anti-Flag antibody and detection of full-length p75 with an anti-p75 antibody. As shown in Figure 3G, wild-type p75 forms a dimer with Flag-ΔECD-p75. In comparison to wild-type p75, the p75 mutant E377R shows a significant decrease in dimer formation, suggesting the importance of this residue and the negative charge in the homodimer interface. In contrast, the mutant H376E has a stronger binding than wild-type p75 (Figure 4E). The role of H376E mutation in dimer formation suggests that the dimer formation could be dependent on the ionization of H376 and then on the pH of the solution. The fact the mutation H376E favors the dimerization suggests that an electrostatic interaction plays a role in homodimerization.

### NgR Interaction Prefers the Presence of p75 Dimers Stabilized by DD and Cys257

**Figure 5:**
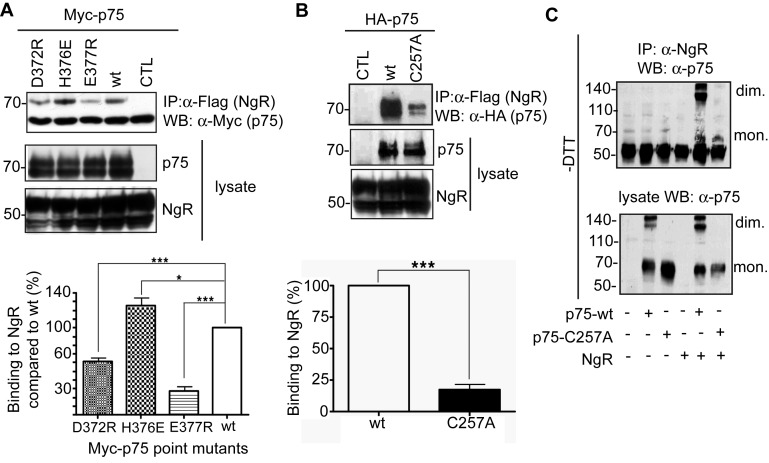
p75 stabilized dimers through both DD and TM domain are required for NgR interaction. (A) Immunoprecipitation experiments of wild-type or mutant p75 with NgR that is Flag-tagged in HEK293 cells. The same mutants described in Figure 4 that are not co-immunoprecipitated with Flag-ΔECD-p75 are not able to be co-immunoprecipitated with Flag-tagged NgR either, suggesting a role of DD dimer stabilization in NgR binding. WT, 100%; D372R, 61.8%±3.64%, *N* = 5; *** *p*<0.0001; H376E, 125.4%±9.13%, *N* = 5; * *p*<0.1; E377R, 27.4%±4.79%, *N* = 5; *** *p*<0.0001. The data can be found in Table S1. (B) Immunoprecipitation experiments of wild-type or p75-C257A with NgR-Flag in HEK293 cells. The p75-C257A interaction with NgR is impaired in comparison to p75 wild type. WT,100%, C257A, 17.67%±4.67%, *N* = 3; *** *p*<0.0001. The data can be found in Table S1. (C) Immunoprecipitation experiments of wild-type or p75-C257A with NgR-Flag in HEK293 cells and nonreducing SDS-PAGE followed by Western blot. The NgR interaction to p75 dimers is preferred to p75 monomers. The presence of dimers (d) and monomers (m) is labeled in the blot.

Very little is known about how p75 and NgR interact from a structural point of view. To shed light on this and to understand how p45 modulates p75/NgR signaling, we first investigated how p75 and NgR interact with each other. We used the following p75 constructs: (1) p75 dimerization mutants E377R, D372R, and H376E (Figure 4), which exhibit a significant reduction or increase of p75 homodimer formation, and (2) p75-C257A, a mutant in the TM domain of p75, which although able to form dimers, is not functional upon NGF binding [30]. When co-transfected, mutants E377R and D372R showed less interaction with NgR (Figure 5A). However, mutant H376E, which promotes p75 homodimer formation, displayed a significant increase in its capability to bind NgR (Figure 5A and Table S1). We then determined whether C257 plays a role in the interaction with NgR. When co-transfected with NgR, the p75-C257A/NgR interaction was markedly impaired, although some interaction was observed (Figure 5B and Table S1). When we performed the co-immunoprecipitation of NgR with p75-wt and ran a nonreducing SDS-PAGE, we found the majority of p75-wts that were co-immunoprecipitated with NgR were in the form of dimers, whereas NgR co-immunoprecipitated a small and similar amount of p75-wt and p75-C257A in the form of monomers (Figure 5C). This suggests that NgR and p75 form a complex that is better stabilized with p75-wt than with p75-C257A. In addition, because p75-C257A is still able to form dimers as inferred by crosslinking [30],[46], these results suggest that p75-wt dimers mediated by C257 have a preferred conformation for binding to NgR.

From these data we conclude that it is not the mere dimerization of p75 but the conformation stabilized by both disulfide bond and DD electrostatic interactions that is preferred for NgR interaction.

### NMR Solution Structure of p45ICD

**Figure 6:**
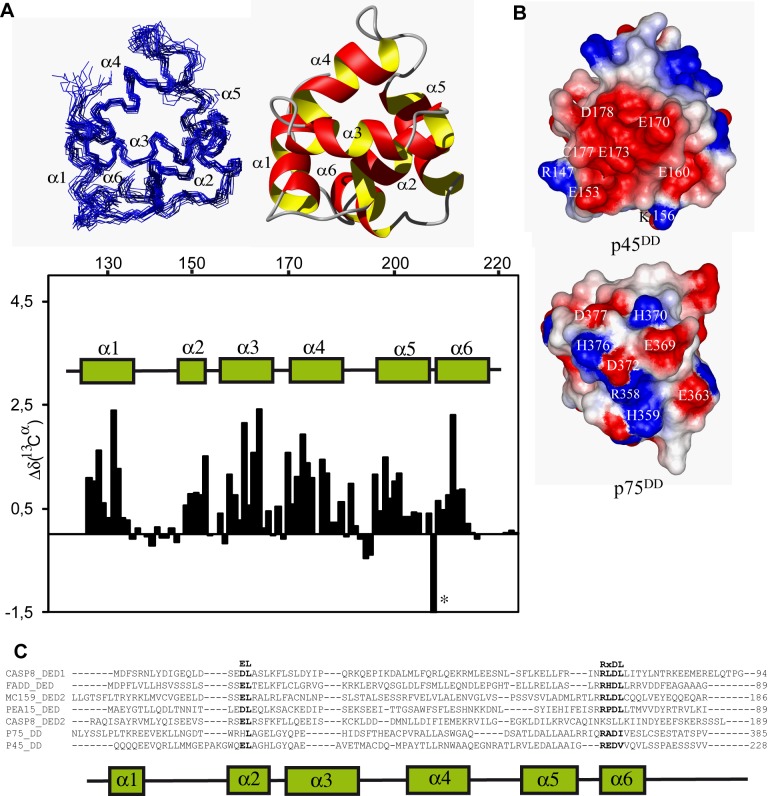
Three-dimensional NMR structure of p45DD. (A) Superposition of 20 conformers representing the 3D NMR structure (left) and ribbon diagram of the lowest energy conformer highlighting the α-helices in red and yellow (right). (Bottom) ^13^C^α^ chemical shift deviation from their corresponding "random coil" values Δδ(^13^C^α^) of p45ICD (residues 130–228). Segments of positive deviations are indicative of helical secondary structure. The location of the six α-helices of p45 are represented by cylinders and labeled accordingly. The asterisk indicates the unusual chemical shift of R211 attributed to the salt bridge between R211 and D213 as well as E160. (B) Electrostatic potential of p45DD and p75DD in a surface representation indicates a highly negative patch around helix α3 of p45DD. The same orientation as in (A) is used. (C) Sequence alignment of DEDs of mouse PEA-15 (Q62048), human FADD-DED (Q13158), human Caspase-8 (Q14790), molluscum contagiosum virus MCV-159 (Q98325), and death domain from rat p75DD (NP_036742) and mouse p45DD (NP_080288). The positions of helices are indicated by the diagram below the p45DD sequence. The conserved motif RxDΦ at the beginning of helix 6 and the conserved residues (EL) in helix 2 are indicated in bold.

Our data (Figure S2) and previous published data from other authors have mapped the interaction between p45 and p75 to the TM and the ICD [27]. To map the binding interface of p45 and p75 ICDs, first we solved the three-dimensional solution structure of mouse p45ICD by NMR spectroscopy (Figure 6). The NMR studies showed that p45ICD contains a flexible domain at the N terminus (residues 75–140) that could not be assigned because they display limited chemical shift dispersions, and a folded domain at the C terminus (141–218) (Figure S7). Figure 6A presents the three-dimensional NMR structure of the folded DD domain obtained from the experimental restraints (Table S2). The regions with a secondary structure are the best defined and typical for a DD, and p45DD is composed of six α-helix disposed in a specific orientation (Figure 6A) comprising residues 141–147 (α1), 154–167 (α2), 169–180 (α3), 182–190 (α4), 200–207 (α5), and 212–218 (α6). A DALI [47] search revealed p75DD as the closest structural relative with an rmsd of 2.7 Å, followed by other members of the DD family (Table S3). The DD of p75 and p45 share many structural features and the same arrangement of all the six α-helices, which is not surprising as p75DD and p45DD are homologues. Only the length of the loop between α1 and α2 is longer in p45DD because of an insertion of four amino acid residues in this segment. When compared with p75DD, the longer loop reorients α1 in respect to α2 and α3 and brings residue E153 of the loop in close neighborhood to other negative charged residues (Figure 6B). Together with some additional amino acid residue differences, this small structural reorientation changes significantly the charge distribution around helix α3 of p45DD (Figure 5B). The negative charged region of p45DD is formed by E153, E160, E170, E173, and D178. In the equivalent region of p75DD (Figure 6B), the negative charged E363, E369, D372, and E377 are located in a more balanced environment surrounded by positive charged residues R358, H370, and H376, which are positively charged depending of their specific pk_a_.

Another interesting feature of the sequence of p45DD is the presence of the RxDΦ motif (x, any residues; Φ, a hydrophobic residue) at the beginning of helix α6 (Figure 5C), which is typically observed in death effector domains (DEDs), such as the DED from PEA-15, FADD, Caspase-8, and others [48],[49]. In DEDs, this motif has been suggested to stabilize the DED fold, because it participates in a salt-bridged network between the arginine side chain and the aspartic acid side chain of the RxDΦ motif, and a glutamic acid side chain located in the helix α2 (for instance, R72, D74, and E19 in FADD-DED) (Figure 6C) [48]. Such a charged network is also present in the three-dimensional structure of p45DD between residues R211, D213, and E160, as indicated by the large downfield shift of the Hε for R211 of p45, indicative of a charged interaction (Figure 6A, the asterisk indicates the downshift of R211). A similar shift has been observed for R72 of FADD-DED [48]. The presence of this salt bridge is an unexpected feature of p45DD, because this motif is not found in any other DD. p75DD has a similar RxDΦ sequence, RADI (highlighted in black in Figure 6C), and from this argument, Park et al. [50] has suggested that p75DD is a DED, not a DD. However, in our hands, we did not see a large shift of the arginine of p75 by NMR, like in p45 and in PEA, suggesting that maybe this arginine is not forming a salt bridge. In fact, in the amino acid sequence of p75, the analogue residue for E160 involved in the salt bridge in p45 is a His residue (H217) (Figure 6C). Nevertheless, the possibility that p45 and p75 DDs are actually DEDs or a chameleon between DD and DED should be considered in future research.

### Mapping the p75DD/p45DD Interface Interaction by NMR

**Figure 7:**
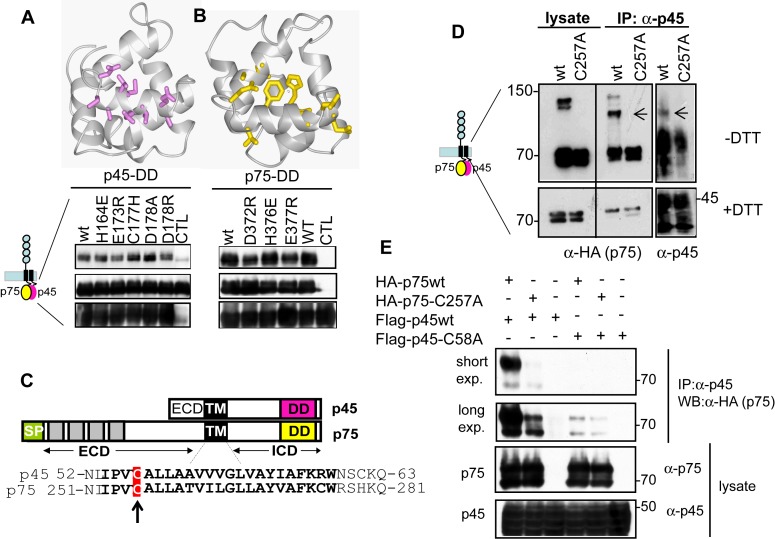
p75/p45 interaction is promoted by DD and TM domain. (A) Co-immunoprecipitation experiments of wild-type or mutant p45 with Myc-p75 in HEK293 cells. p45 mutants show differential binding to p75. The p75DD-dependent chemical shift changes of p45DD are mapped onto the 3D structure of p45DD. (B) Co-immunoprecipitation experiments of wild-type or p75 mutants with Flag-p45 in HEK293 cells showed differential binding to p45. The p45DD-dependent chemical shift changes of p75DD are mapped onto the 3D structure of p45DD. (C) Protein sequences of p75 and p45 TM domains are highly conserved. The conserved cysteine residue is highlighted in a red box. (D) The cysteine residues in the TM domain of both p75 and p45 form a covalent disulfide dimer between p75 and p45. Co-immunoprecipitations of either p75 wild-type or the p75 TM domain mutant (p75-C257A) with p45 wild type were analyzed in HEK293 cells and in reducing and nonreducing SDS-PAGE followed by Western blot. p75 and p45 form a heterodimer sensitive to DTT (arrow). (E) Co-immunoprecipitations of either p75 wild type or the p75 TM domain mutant (p75-C257A) with p45 wild type or p45 C58A mutant were analyzed in HEK293 cells, indicating that both p75-C257A and p45-C58A TM domain mutants diminish the interaction between p75 and p45.

We then started the characterization of the p75ICD–p45ICD interaction. Using NMR chemical shift perturbation experiments with p45ICD, the binding site of p75ICD on p45ICD was mapped to α2/α3 in the DD of p45 (Figure S8). To further characterize the interaction between p45DD and p75DD in the corresponding full-length proteins, p45 point mutations at some residues with significant chemical shift changes were constructed (i.e., H164E, E173R, C177H, D178A, and D178R). The presence of a full-length p45–p75 complex in correspondingly transfected HEK293 cells was measured by co-immunoprecipitation of p75 with wild-type or mutant p45 (Figure 7A). Wild-type p45 formed a complex with p75. Of the entire series of p45 mutant constructs studied, E173R and D178R showed a significant decrease in complex formation, whereas C177H and D178A showed a significant increase in complex formation (Figure 7A). NMR experiments with purified p45 mutants showed that they were well folded (Figure S9). These data are consistent with the structural studies and suggest that p45 forms a complex with p75 through its helix α3 of the DD. Next, the amino acid residues in p75ICD that interact with p45ICD (Figure S4) were characterized. In the chemical shift perturbation experiment, p45ICD is titrated against 10 µM of ^15^N-labeled p75DD. Chemical shift changes on p75DD are observed in amino acid residues located close to and on helix α3 of p75DD (i.e., L360, E363, Q367, H370, D372, F374, T375, H376, E377, A378, A383, L384, L385, W388), indicating helix α3 is the binding site for p45DD on p75DD (Figure 7B). The effect on the interaction between p45 and p75 by some of the amino acid replacements located in this side of p75DD (D372R, H376E, and E377R) supports the NMR-derived interface (Figure 7B). A comparison of the p45DD binding site on p75DD (Figure 7A) with the p75DD homodimer binding site (Figure 4D) shows that the two sites overlap. The presence of an overlapping binding site on p75 suggests that p45 is binding to a monomeric p75 by forming a heterodimer. We tried to purify a stable complex between p75ICD and p45ICD by gel filtration, but we were unsuccessful. It could be possible that p45ICD interaction inhibits the formation of p75 dimers or multimers, but the interaction is too weak to yield sufficient p75/p45 heterodimers.

### p45DD Forms a Heterodimer with p75DD by Breaking the p75DD Dimer

The presence of a partially overlapping binding site on p75DD for homodimerization with p75DD and heterodimerization with p45DD suggests that p45DD may break a p75DD dimer by competition. To get insights into this potential mechanism, the p45ICD-induced chemical shift perturbations of p75ICD were measured at high (dimeric) and low (monomeric) concentrations of p75ICD. p45ICD binds p75ICD even at high p75ICD concentrations, where p75ICD is mainly homodimeric (Figure S6). As demonstrated above, A378 is participating in the homodimer interface of p75DD because chemical shift differences between low and high concentrated p75ICD were observed (Figure S10). However, the addition of p45ICD at low concentrations of p75ICD did not result in any chemical shift changes of A378, indicating that A378 is not part of the p45–p75 binding site. In contrast, the addition of p45ICD at a high (dimeric) concentration of p75ICD generated chemical shift perturbations of the ^15^N-^1^H moiety of A378 to values identical to p75ICD at a low (monomeric) concentration in absence of p45ICD (Figure S10). Our explanation of these findings is as follows. Although A378 is participating in p75DD homodimer formation, it is not involved in p45DD binding. However, the presence of p45 breaks the p75 dimer and forms a p45–p75 heterodimer and thus shifts A378 from a dimeric environment to a monomeric environment (Figure S10).

Complementary information about the breaking of a p75 homodimer into a p45–p75 heterodimer can be extracted by the p75ICD- and p45ICD-dependent chemical shift perturbations of T375. T375 has different chemical shifts at high and low concentrations of p75ICD, indicative of its participation in the p75DD homodimer formation. However, upon the addition of p45DD, the chemical shifts of T375 move to a new position that is independent of the p75ICD concentration (at least at the concentration window studied here; Figure S10). This result suggests that T375 is involved in both p45DD and p75DD binding. Furthermore, the data indicate that p45 is able to compete with the p75DD homodimer by the formation of a p45DD–p75DD heterodimer. Other residues of p75 showed similar behavior, suggesting that p45DD is able to break the relatively weak p75DD homodimer by forming a heterodimer p45DD–p75DD.

### p75 and p45 Heterodimerize Through Their TM Domain by p75–C257/p45–C58 Interaction

The results from above suggest that the p45 modulation of p75 signaling is increased with the presence of the TM domain of p45. The TM domain of p75 self-associates [30]. Because the TM domain of p45 is highly homologous to p75-TM (Figure 7C), we asked if p45-TM was able to bind p75 through its TM. Co-immunoprecipitation experiments were conducted in HEK293 cells transfected with p75 wt and p45, and analysis in nonreducing SDS-PAGE showed a band recognized by both p75 and p45 antibodies and with a molecular weight corresponding to a heterodimer p75–p45 (Figure 7D). This band was lost when p75-C257A was co-transfected with p45. These results suggest that p75 and p45 can form a heterodimer through C257 from p75 and another cysteine residue from p45. Due to the homology in the TM domain, we mutated the cysteine equivalent in p45, Cys58 (Figure 7C). As shown in Figure 7E, p45-C58A did not form a complex with wt p75. Only when the blot was exposed longer did we see an interaction between p45 and p75, presumably by interaction through their intracellular DDs.

## Discussion

**Figure 8:**
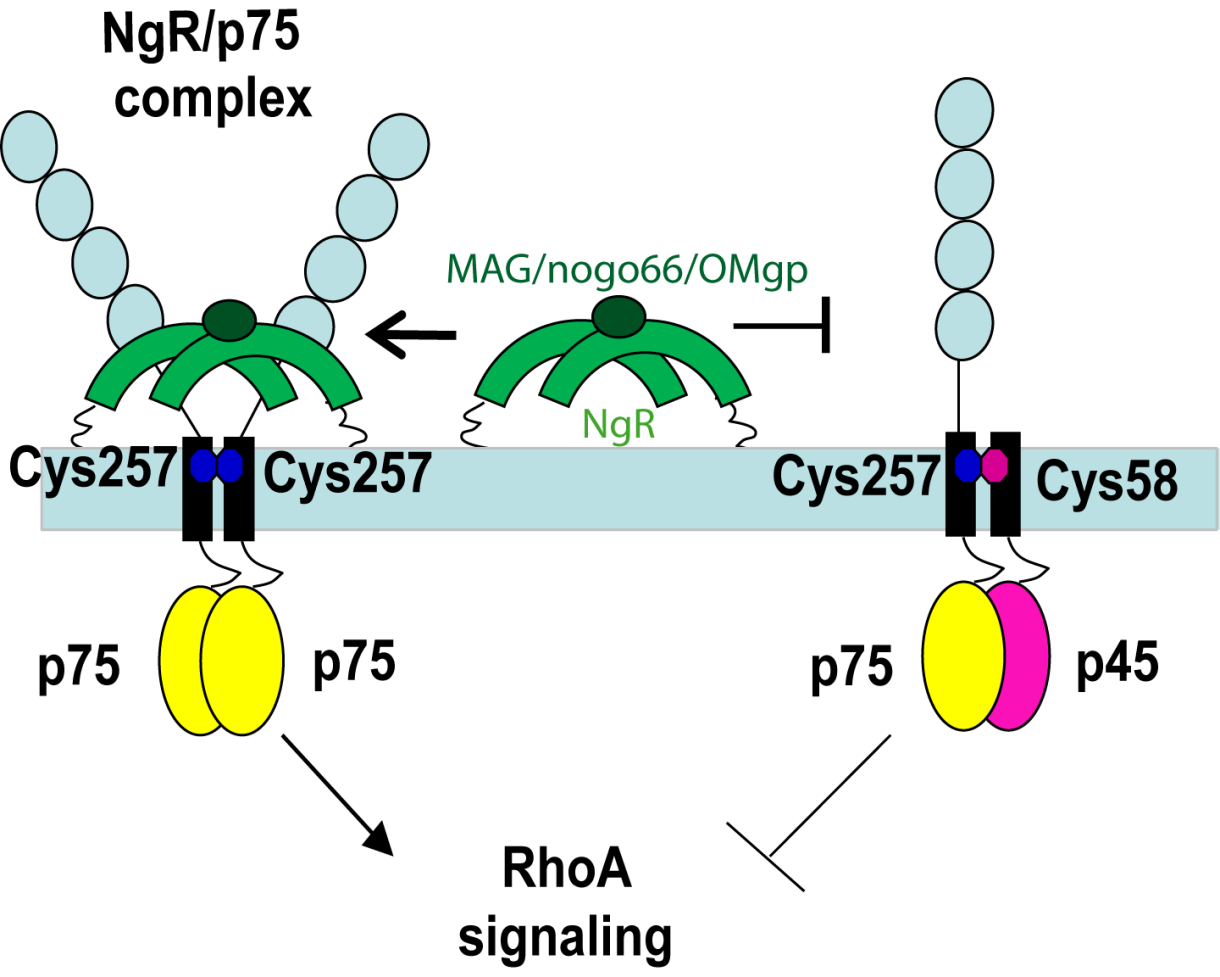
Model illustrating p45 inhibition of p75/NgR signaling. p75 is a constitutive dimer in the membrane, stabilized by the Cys257 at the TM domain, where it can bind to NgR complex and activate RhoA signaling and axonal growth collapse. When p45 binds to p75 through both the TM domain (Cys257–Cys58) and DD interactions, p75 downstream signaling is inhibited.

The presented biophysical and biological characterization of p45 and p75 interactions shows that a p45–p75 heterodimer is formed, using both the TM and the ICDs. p45 binding to p75 inhibits RhoA activation and increases neurite outgrowth. In light of these data, the following mechanistic model of p45 action is proposed (Figure 8). NgR binding to p75 recruits intracellular proteins that activate RhoA signaling. In the presence of p45, however, p45–p75 heterodimers are formed, stabilized by the Cys257–Cys58 interaction within the TM domain and enhanced by the interaction of cytoplasmic domains. In this heterodimer conformation, the interaction of NgR with p75 is impaired and this translates into diminished RhoA signaling.

p45 interaction with p75 has been described previously [23],[27]. However, a prominent role of the TM domain in that interaction was not suggested. The interaction between p45 and p75 is decreased by a deletion of the p75ICD, but not entirely blocked, suggesting that the TM domain also has a prominent role in the p75/p45 interaction (see figure 3C in [27]). It is also interesting to note that p45 has been suggested previously to interact with TrkA and modulate its activity [25]. In that publication, the authors made several deletion constructs of p45 and suggested that the TM domain of p45 was needed for the p45–TrkA interaction [25],[51]. That observation, together with our data, suggests that the TM domains of neurotrophin receptors are starting to be recognized as very important for their function.

One important question that remains to be answered is where and how in the cell the p45/p75 heterodimer could be formed. It is difficult to imagine that p45 could break a covalently formed p75 homodimer, however free monomeric p75 is present in the cell membrane [30]. One possibility is that the heterodimer is formed in the ER during the translation and maturation of both proteins. Kim et al. have shown that p45 is involved in the trafficking of sortilin to the plasma membrane, indicating that the p45/sortilin interaction takes place in the ER membrane [27]. The p75/p45 interaction could also occur in the ER membrane during receptor maturation. This implies that it requires the synthesis of new proteins for p45 activity, and only when new p75 and p45 molecules are synthesized will they have the opportunity to form the heterodimers. Such a situation is plausible because both p75 and p45 are up-regulated upon nerve injury and they are expressed in the same cells (Figure S11). In that situation, at the plasma membrane, it could be possible to have different oligomers of p75—namely, p75 monomers, p75 homodimers, and p75/p45 heterodimers. The function of those species may be different, contributing to the complexity of p75 signaling.

We showed that p75ICD exists in a monomer–dimer equilibrium mediated by electrostatic interactions, and we postulate that p75 dimerization is pH-dependent or promoted by the presence of counterions, like phosphate in our buffer. This could reconcile the contradictory results reported by previous studies of p75ICD structure. NMR structural studies detected only the monomeric p75ICD species at a pH in the range of 6–7 and in plain water with no counterions [45], solution conditions in which p75ICD will not be favored to self-associate. In contrast, X-ray structures of p75ICD revealed a dimer [41], but the buffers used for crystallization were at or above pH 7.0 and contained 1.1–1.4 M sodium malonate or sodium citrate, as stabilizing counterions. Thus, we conclude that p75 dimerization is dependent on the pH and the presence of counterions.

It is interesting to compare the homo- and hetero-dimeric interactions found here with other protein oligomerizations that involve DDs and DEDs. Although DDs and DEDs were first identified in proteins that mediate programmed cell death, they are now recognized to act as protein interaction domains in a variety of cellular signaling pathways [28]. In the DD subfamily, low sequence homology produces diverse interaction surfaces, enabling binding specificity within a subfamily [52]. Protein–protein interactions of DDs have been thereby thought to be predominantly homotypic among different adaptors, as shown here for p75DD homodimer formation, although some examples of heterotypic interactions have been demonstrated (reviewed in [28]), including the p45–p75 presented here. These results suggest that DDs employ diverse mechanisms for interactions [53]. They can be classified into three types of interactions (reviewed in [52]). Type I interaction is exemplified by the procaspase-9 CARD:Apaf-1 CARD complex [54], whereas the type II interaction is represented by the Pelle DD:Tube DD complex [53], and the type III interaction is proposed to exist in the Fas DD:FADD DD complex [55]. p75 and p45 DD interaction appears symmetrical based on chemical shift data, but one cannot exclude an asymmetrical interaction that uses the same regions of the interface; as p75 is a homodimer and p45 and p75 binding sites overlap only in parts, the p45–p75 interaction could not be totally symmetric. Because p75DD interacts with itself through residues located between helices α-3 and the loop connecting α-3 and α-4, they belong to a type III interaction [48]. The asymmetric type I, II, and III interactions between DDs are conserved in all current structures of oligomeric DD signaling complexes [55]–[57]. These interactions likely represent the predominant mechanism of DD polymerization.

Here, p45DD acts as an inhibitor of those interactions, shutting off or modulating the p75 signal strength. Interestingly, both p45DD and p75DD are promiscuous and can interact also with other proteins through their DD. Although p75DD is able to interact with downstream targets, such as the CARD of RIP-2 [58], p45DD appears to interact with FADD, thereby reducing FADD-mediated cell death [29]. Recently the regions of p75DD involved in the three different p75 signaling paradigms has been mapped by mutagenesis—namely, apoptosis, NF-κB activation, and Rho signaling [59]. For p75/p45 heterodimers, p45DD will occupy the region very close to where the CARD domain of RIP-2 is binding to p75DD, according to previous data [59]. Strikingly, RIP-2 binding to p75 is necessary for Rho-GDI release and RhoA inhibition. The data suggest that p45DD binding to p75 will release RhoGDI and inhibit RhoA activation. This is in agreement with our data that p45 binding to p75 inhibits RhoA activity.

Recently it has been described that p75 could adopt two different conformations, a symmetrical dimer, stabilized by a cysteine disulfide bond, and an asymmetrical dimer [41]. The authors proposed that p75 could be in equilibrium between both conformations unless oxidant conditions inside the cell promote the formation of the disulfide bond [41]. Our NMR data suggest that the symmetrical conformation is the predominant form at least in solution, because interaction between residues from helix 3 and helixes 5–6 are not seen in our conditions. Further investigation will be needed to understand which conformation belongs to the active receptor.

The fact that p45 is able to bind and to block the symmetrical interface suggest a well-designed and potent p75 inhibitor. Apart from the p75 signaling, p45 might play additional roles of an inhibitory nature. In particular, because the DD of p45 appears to be important for p45/p75 interaction and several members of the TNFR family contain DDs such as TNF-R1 or CD95, which have been shown to play important roles in SCI [60],[61], it is intriguing to speculate whether p45 may antagonize the activity of some of these receptors upon SCI by binding to their DD domains as well. Thus, the promiscuous structural nature of p45 may facilitate functional recovery after SCI by inhibiting multiple signaling pathways that are detrimental for neuronal survival and nerve regeneration.

In summary, p45 presents an example of a new antagonizing mechanism by which an interaction mediated by the TM and cytoplasmic domains is able to inhibit p75 disulfide dimer formation and function. Such knowledge provides a new glimpse into our understanding of the multiple distinct activities and signaling capabilities of p75.

## Materials and Methods

### Ethics Statement

In this study, animal protocols were approved by the Institutional Animal Care and Use Committee (IACUC) of the Salk Institute and the Council on Accreditation of the Association for Assessment and Accreditation of Laboratory Animal Care (AAALAC International). Euthanasia will be performed by methods specified and approved by the IACUC Panel on Euthanasia.

### Antibodies

The following antibodies were used in the present studies. Anti-p75 antibodies (anti-p75ECD epitope: 9651, a gift from Dr. Moses Chao, New York University; anti-p75ICD epitope: Buster, a gift from Dr. Phil Barker, Montreal Neurological Institute), anti-p45 antibodies (anti-p45ECD epitope, 6750 and antip45ICD epitope, 6655 were generated in house), and anti-V5 antibody were purchased from Life Technologies (R960-25); anti-Falg M2 antibody was purchased from Sigma (F1804); and anti-HA antibodies were purchased from Roche (clone 3F10, 11867423001) and Santa Cruz Biotechnology (sc-805).

### Protein Construct Expression and Purification

For the recombinant expression of p45ICD, *Escherichia coli* BL21(DE3) freshly transformed with the pGST-p45 expression vector, which encodes a N-terminal GST tag and a thrombin cleavage site, was used. Two liters of M9 minimal medium containing either (NH_4_)_2_SO_4_ or (^15^NH_4_)_2_SO_4_ and either glucose or ^13^C-glucose as the sole nitrogen and carbon sources were inoculated in the presence of ampicillin (100 µg/ml) with 30 ml of preculture of pGST-p45–containing *E. coli* BL21(DE3) cells, which had been grown at 37 °C overnight. At OD_600_ = 0.6, expression was induced with 1 mM isopropyl L-D-galactopyranoside. The cells were harvested after 4 h, and the pellet was resuspended in 30 ml of PBS. After sonication, the cell lysate was centrifuged at 20,000 *g* for 30 min, and the supernatant was incubated with Glutathione Sepharose (Amersham) in PBS with DTT 1 mM at 4 °C for 1 h. After several washes with PBS, the GST fusion protein was removed by thrombin digestion overnight at room temperature while still bound to the sepharose resin. The digest was incubated with benzamidine resin to remove the thrombin (Sigma). The supernatant contained free p45ICD with 95% purity according to SDS-PAGE.

For the recombinant expression of human p75ICD, *E. coli* BL21 (DE3) freshly transformed with the pHisp75 expression vector, which encodes a N-terminal 22-aa affinity tag containing a six-histidine sequence and a thrombin cleavage site, was used. Two liters of unlabeled or stable-isotope–labeled minimal medium (see above) containing kanamycin (50 µg/ml) were inoculated with 30 ml of preculture of pHisp75-containing *E. coli* BL21(DE3) cells that had been grown at 37 °C overnight. At OD_600_ = 0.6, expression was induced with 1 mM isopropyl L-D-galactopyranoside. The cells were harvested after 4 h, and the pellet was resuspended in 30 ml buffer A (25 mM Tris-HCl, pH 8.0/500 mM NaCl/10 mM β-mercaptoethanol). After sonication, the cell lysate was centrifuged at 20,000 *g* for 30 min, and the supernatant was applied to a Ni2+-charged NTA column (Qiagen, Chatsworth, CA). The fusion protein was eluted with a stepwise gradient of 0–500 mM imidazole in buffer A. After dialysis against PBS (pH 8.0), the N-terminal fusion tail was removed by thrombin cleavage performed as described above. The supernatant contained free p75ICD with 95% purity according to SDS-PAGE. The mutant p75 constructs were expressed and purified accordingly.

The protein constructs were concentrated using a 10 kDa centriprep amicon concentrator. The concentrations of all proteins used in this study were determined from their absorbance at 280 nm by using molar extinction coefficients calculated from the Expasy protein server software (http://www.expasy.ch). If not stated otherwise, all biophysical experiments were measured in PBS pH 8, 100 mM NaCl.

### Size Exclusion Chromatography

Ni-NTA purified p75ICD and GST-sepharose purified p45ICD were loaded on an S200-Superdex gel filtration column at 4 °C and eluted isocratically in PBS pH 8.0 buffer at 0.7 ml/min.

### Analytical Ultracentrifugation

Sedimentation equilibrium measurements of samples of p75ICD and p45ICD were conducted at concentrations of 10 µM, 300 µM, and 700 µM. Data were collected at four different speeds (10,000, 14,000, 20,000, and 28,000 rpm).

### NMR Spectroscopy

The NMR experiments were carried out on a Bruker DRX700 spectrometer at 25 °C by using protein solutions that contained PBS pH 8.0, 100 mM NaCl, 1 mM sodium azide, and 95%/5% H_2_O/D_2_O. Sequential assignment and structure determination was performed with the standard protocol for ^13^C, ^15^N-labeled proteins [62]. Hence, sequential assignments of backbone resonances of ^15^N,^13^C-labeled p45ICD and ^15^N,^13^C-labeled p75ICD were obtained from HNCA^coded^CB, HNCA^coded^CO [63]. HNCA [42] and ^15^N-resolved [64] NOESY [65] spectra. The side chain signals of p45ICD were assigned from HCCH-COSY [66] and ^13^C-resolved [64] NOESY experiments. Aromatic side chain assignments were obtained with 2D [64] NOESY in D_2_O [64]. Distance constraints for the calculation of the 3D structure were derived from 3D ^13^C-,^15^N-resolved [64] NOESY and 2D [64] NOESY spectra recorded with a mixing time of 80 ms. [42]-TROSY [43] spectra with parameters as described below were measured for p75ICD mutants. The data were analyzed using the CARA software program (www.nmr.ch).

### Chemical Shift Perturbation Experiments

For the chemical shift perturbation experiments, [42]-TROSY spectra [43] of stable isotope-labeled p45ICD or p75ICD were measured with t_1,max_ = 88 ms, t_2,max_ = 98 ms, and a data size of 200×1,024 complex points. [42]-TROSY experiments of ^13^C,^15^N-labeled p45ICD were performed at protein concentrations of 0.1 mM and 0.5 mM in order to study the oligomerization state of p45ICD. [42]-TROSY experiments of ^15^N-labeled p75ICD were performed at protein concentrations of 10 mM, 0.1 mM, 0.2 mM, 0.5 mM, and 2 mM to study the oligomerization state of p75ICD and to elucidate the homodimer interface. [42]-TROSY experiments of ^15^N-labeled p75ICD were performed at a protein concentration of 10 mM free and in the presence of 0.1 mM unlabeled p45ICD to study the p45ICD–p75ICD heterodimer interface. [42]-TROSY experiments of ^15^N-labeled p75ICD were performed at a protein concentration of 0.5 mM at a 1:0 mixture of ^15^N-labeled p75ICD and unlabeled p45ICD followed by stepwise addition of unlabeled p45ICD up to a p45ICD concentration of 2 mM (protein ratios were 1:0, 1:0.5, 1:1, 1:2, and 1:4). The same NMR setups were used in titration experiments performed to investigate the binding site of p75ICD on p45ICD. The titration experiments were started with a 1:0 mixture of ^13^C,^15^N-labeled p45ICD, and unlabeled p75ICD was added stepwise from 0 mM to 2 mM (protein ratios were 1:0.5, 1:1, 1:2, and 1:4).

### Structure Calculation

We observed 2,130 NOEs in the NOESY spectra, leading to 1,065 meaningful distance restraints and 372 angle restraints (Table S2). For the structure calculation, the program CYANA was used [67],[68], followed by restrained energy minimization using the program INSIGHT. CYANA initially generated 100 conformers, and the 20 conformers with the lowest energy were used to represent the three-dimensional NMR structure. The 20 refined conformers showed small residual constraint violations that are compatible with the observed NOEs and the short interatomic distances (Table S2). Similar energy values were obtained for all 20 conformers. The quality of the structures is reflected by the RMSD values of 0.65 Å relative to the mean coordinates of p45 residues 141–218 (see Table S2 and Figure 5A). The bundle of 20 conformers representing the NMR structure is deposited in the PDB database under accession no. 2IB1.

### Transfection and Immunoprecipitation

Constructs containing NgR, full-length p45 or p75, as well as deletion and amino-acid point mutants were transfected into HEK293 cells by TransFectin transfection reagents (BioRad). Transfected cells were collected and lysed in RIPA buffer (150 mM NaCl, 1% NP-40, 0.5% DOC, 0.1% SDS, 50 mM Tris pH 8.0). Lysates were immunoprecipitated with antibodies described in the text. Samples were analyzed using SDS-PAGE and Western blots. For quantitative analysis, gel images were analyzed by Image J program (NIH). Statistical analyses and data graph were done using Prism software.

### RNA Transfection and Neurite Outgrowth Assay

Full-length p45 RNA with capping at the 5′ end and poly(A) sequences was transcribed *in vitro* using the mMessage-mMACHINE kit (Ambion). RNA was transfected into CGNs with the TransMessenger transfection reagent (Qiagen). We found that this protocol achieves 50%–70% transfection efficiency. Neurite outgrowth assay using CGNs was carried out as previously described [14].

### RhoA Assay

CGN culture was performed as previously described [13],[14]. Briefly, p5–p7 cerebella were isolated from WT and Thy1-p45 mice. Neurons were plated on Poly-D-Lysine–coated six-well tissue culture dishes at the density of 5 million cells per well. Cells are allowed to grow for 48 h and then starved in basal medium eagle (BME, Life Technologies) for 7 h before being treated with preclustered MAG-Fc at the concentration of 2 µg/ml for 15 min. Preclustered human IgG was used as the control. The preclustering is achieved by incubating the MAG-Fc with an anti-human-Fc antibody at a 2:1 molar ratio in BME for 30 min in 37 °C. Cells are then lysed on ice according to the manufacturer's suggestion, and the RhoA assay was performed following the instructions of the G-LISA (absorbance based) kit. The RhoA activity is measured by reading the 490 nm absorbance using a 96-well plate reader. We have also used the Millipore (Upstate) RhoA assay kit for a pull-down of activated RhoA by Western blotting analysis.

## Supporting Information

Figure S1 p45 does not bind to NgR. A plasmid expressing V5-tagged p45 or V5-tagged p75 were co-transfected in 293T cells with a plasmid encoding for Flag-NgR. Western blots show that p75 and NgR interact upon co-transfection and co-immunoprecipitation with Flag antibody (M2). However, p45 does not co-immunoprecipitate with Flag-NgR, suggesting p45 modulates p75/NgR signaling through p75, not directly interacting with NgR.
(TIF)

Figure S2 The inhibition of p75/NgR interaction by p45 requires the TM and ICD domains of p45. Different p45 deletion mutants were co-transfected with p75- and hNgR-expressing vectors. p45 devoid of the ECD has similar blocking activity as the full-length p45. In contrast, constructs without the ICD or TM display a much lower blocking activity. CTL, 100%; *** *p*<0.0001; p45-FL, 54.25±7.69, *N* = 4, compared to p45-ECD-TM, 65.25±4.69, *N* = 4; unpaired *t* test, ns; *F* test, ns. p45-ICD, 67±4.55, *N* = 4, compared to p45-TM-ICD, 45.5±5.78, *N* = 4, unpaired *t* test: * *p*<0.1; *F* test, ns. p45-ECD-TM, 65.25±4.69, *N* = 4, compared to p45-TM-ICD, 45.5±5.78, *N* = 4, * *p*<0.1. The data can be found in Table S1.
(TIF)

Figure S3 Overexpression of p45 inhibits MAG-Fc–induced RhoA activation. (A) Increased p45 protein levels in P5–P7 CGNs following transfection of p45 RNA. (B) Following transfection with the p45 RNA, GCNs were treated with MAG-Fc and subjected to a RhoA activity assay. Overexpression of p45 inhibited MAG-Fc–induced RhoA activation.
(TIF)

Figure S4 Iodoacetamide purification of p75-ICD. SDS-PAGE in reducing and nonreducing conditions of p75-ICD purified from *E. coli* using iodoacetamide, a blocking agent of free cysteines, in the lysis buffer. The absence of dimerized p75-ICD in these conditions suggests that p75-ICD dimerization is produced during the purification as a result of oxidation of free cysteines.
(TIF)

Figure S5 p75-ICD covalent disulfide dimmer formation in the presence of hydrogen perxiode. Nonreducing and reducing SDS-PAGE of purified p75-ICD from *E. coli* was incubated with hydrogen peroxide (10 mM) during the indicated time points. Note that p75-ICD purified from *E. coli* (without DTT, t = 0 min) has already some amount of disulfide dimer.
(TIF)

Figure S6 p75-Cys379 is responsible for disulfide dimerization of p75-ICD. Gel filtration profile of purified p75-ICD WT in reducing (blue) and nonreducing conditions (dark blue) and of purified p75-C379S (green). The elution of p75-C379S is indicative of a monomeric p75-ICD.
(TIF)

Figure S7 Summary of NOEs. Observed NOEs are summarized for p45ICD. Sequential NOEs are indicated by thick horizontal bars. The thickness of the bar is proportional to the magnitude of the NOE intensity. Thin horizontal bars indicate long-distance NOEs.
(TIF)

Figure S8 Insights into p45ICD-p75ICD heterodimer formation. p45ICD-dependent chemical shift changes versus the amino acid sequence observed in stable isotope-labeled p75ICD at (B) 10 µM and (C) 2 mM p75ICD concentration. The bar plot represents the normalized change of the chemical shifts of p75ICD in the absence and presence of p45ICD in the [^15^N,^1^H]-TROSY spectrum using the equation N = 25[Δ(δ(^1^H))^2^ + Δ(δ(^15^N))^2^]^0.5^, where δ(^1^H) and δ(^15^N) are the chemical shifts in part per million (ppm) along the ω_2_(^1^H) and ω_2_(^15^N) dimensions, respectively. Perturbations larger than 0.2 ppm are labeled.
(TIF)

Figure S9 2D-NMR of selected ^15^N-labelled p45-ICD mutants expressed in *E. coli*. p45 DD mutants were expressed and purified as ^15^N-labelled proteins and analyzed by NMR spectra, indicating that all p45 mutants are correctly folded like p45-WT.
(TIF)

Figure S10 Insights into p45–p75 heterodimer formation from NMR. (A) NMR analysis of p45-DD and p75-DD interaction suggests a heterodimer formation. A378, red colored lines represent the cross-peak of A378 in the [^15^N,^1^H] TROSY spectrum at a high concentration of p75ICD (homodimer), and the corresponding cross-peak at a low concentration (monomer) of p75ICD is represented by green dashed lines, respectively. Upon p45ICD addition to a highly concentrated p75ICD sample, the cross-peak of A378 colored as blue lines shifted to the position of the monomer. In contrast, upon p45ICD addition to the sample with low p75ICD concentration, the cross-peak of A378—represented as blue dashed lines—did not shift. These findings indicate that A378 is not part of the p45–p75 interface and that p45 breaks the p75 homodimer. T375, the cross-peaks of T375 in the [^15^N,^1^H]-TROSY spectra at high and low p75ICD concentrations and in the presence and absence of p45ICD are displayed with the same color code as for A378. The addition of p45ICD at high p75ICD concentrations results in a shift of the cross-peak of T375 (red cross-peak to blue cross-peak). The cross-peak of T375 at low p75ICD concentrations is also shifted upon addition of p45ICD (cross-peak represented by green dashed lines to cross-peak represented with blue dashed lines). Because the position of the cross-peak of T375 in the presence of p45 is independent of the p75ICD concentration, T375 appears to be part of the p45–p75 interface as well as part of the p75–p75 interface.
(TIF)

Figure S11 Co-localization of p75 and p45 expression in lumbar spinal motorneurons after sciatic nerve crush. Confocal images of immunofluorescence staining of spinal cord sections from mice with the sciatic nerve crush. (A) p75 staining, (B) p45 staining, and (C) merge of (A) and (B). Some p75-expressing motor neurons also express p45.
(TIF)

Table S1 Data for Figures 2, 5, and S2.
(XLSX)

Table S2 NMR and structural statistics of the 3D structure of p45-DD.
(DOCX)

Table S3 Structural homology search with DALI Server showing the top 10 matches.
(DOCX)

## Abbreviations

CGNs: cerebellar granule neurons
DD: death domain
ECD: extracellular domain
ICD: intracellular domain
MAG: myelin associated glycoprotein
NgR: Nogo receptor
NMR: nuclear magnetic resonance
NOE: nuclear Overhauser effect
NRADD: neurotrophin receptor alike DD protein
NRH2: neurotrophin receptor homologue 2
PLAIDD: p75-like apoptosis inducing DD protein
TM: transmembrane
TNFR: tumor necrosis factor receptor
