# Supplementary figures and images for: Heterodimerization of p45–p75 Modulates p75 Signaling: Structural Basis and Mechanism of Action
Source: PLoS Biol. 2014 Aug 5;12(8):e1001918. doi: 10.1371/journal.pbio.1001918 (PMC4122344; doi:10.1371/journal.pbio.1001918)

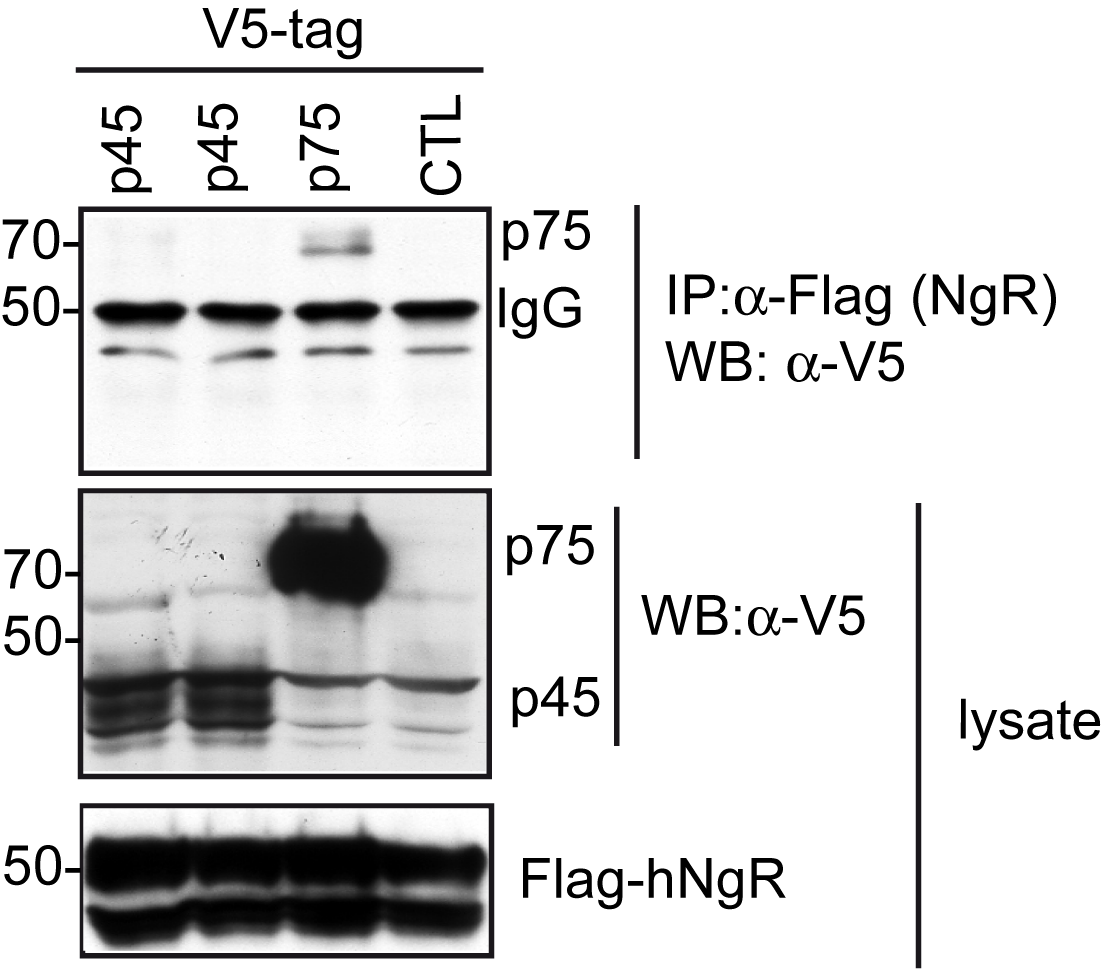

Supplement: Figure S1 — p45 does not bind to NgR. A plasmid expressing V5-tagged p45 or V5-tagged p75 were co-transfected in 293T cells with a plasmid encoding for Flag-NgR. Western blots show that p75 and NgR interact upon co-transfection and co-immunoprecipitation with Flag antibody (M2). However, p45 does not co-immunoprecipitate with Flag-NgR, suggesting p45 modulates p75/NgR signaling through p75, not directly interacting with NgR. (TIF) [file pbio.1001918.s001.tif]

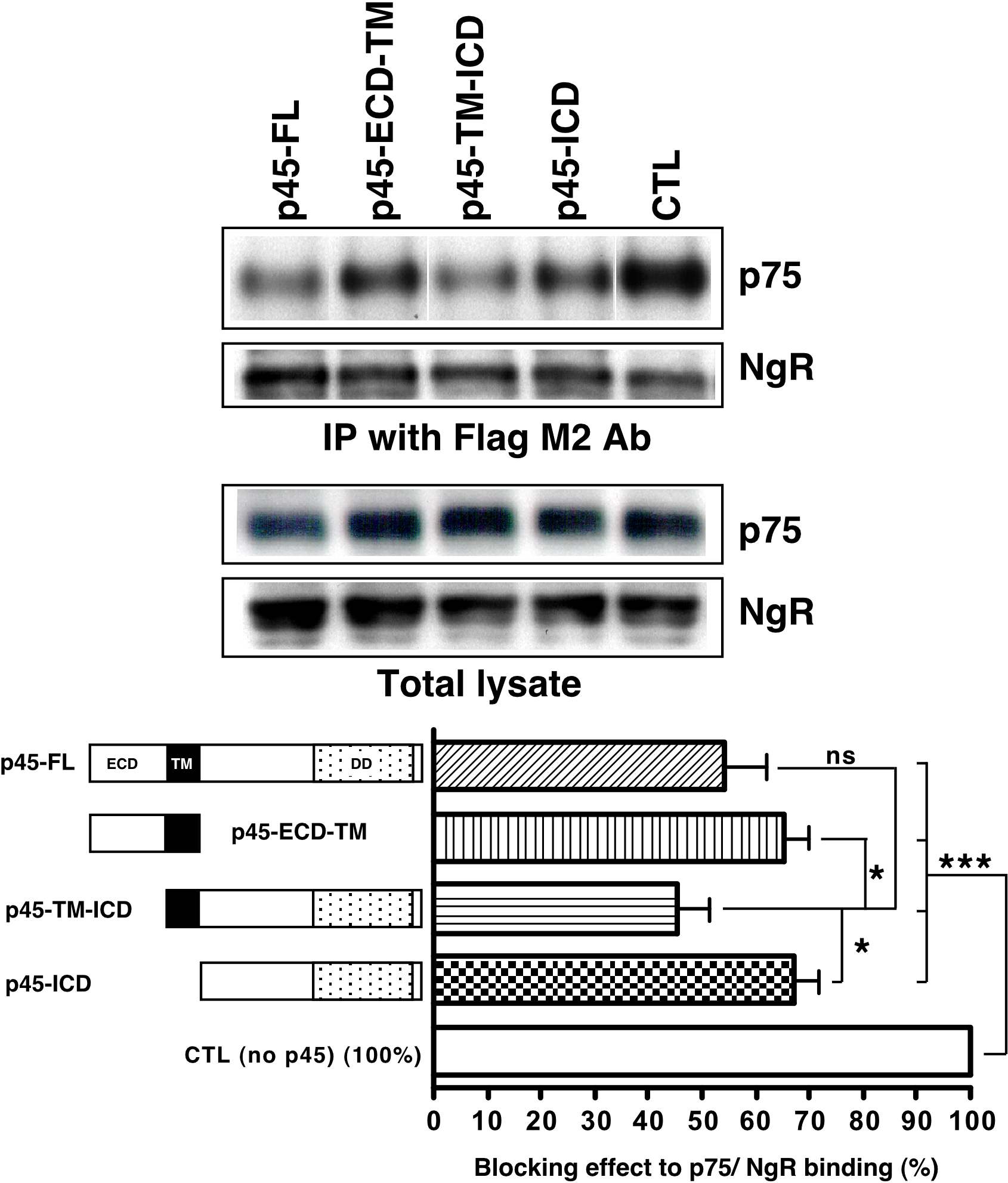

Supplement: Figure S2 — The inhibition of p75/NgR interaction by p45 requires the TM and ICD domains of p45. Different p45 deletion mutants were co-transfected with p75- and hNgR-expressing vectors. p45 devoid of the ECD has similar blocking activity as the full-length p45. In contrast, constructs without the ICD or TM display a much lower blocking activity. CTL, 100%; *** p<0.0001; p45-FL, 54.25±7.69, N = 4, compared to p45-ECD-TM, 65.25±4.69, N = 4; unpaired t test, ns; F test, ns. p45-ICD, 67±4.55, N = 4, compared to p45-TM-ICD, 45.5±5.78, N = 4, unpaired t test: * p<0.1; F test, ns. p45-ECD-TM, 65.25±4.69, N = 4, compared to p45-TM-ICD, 45.5±5.78, N = 4, * p<0.1. The data can be found in Table S1. (TIF) [file pbio.1001918.s002.tif]

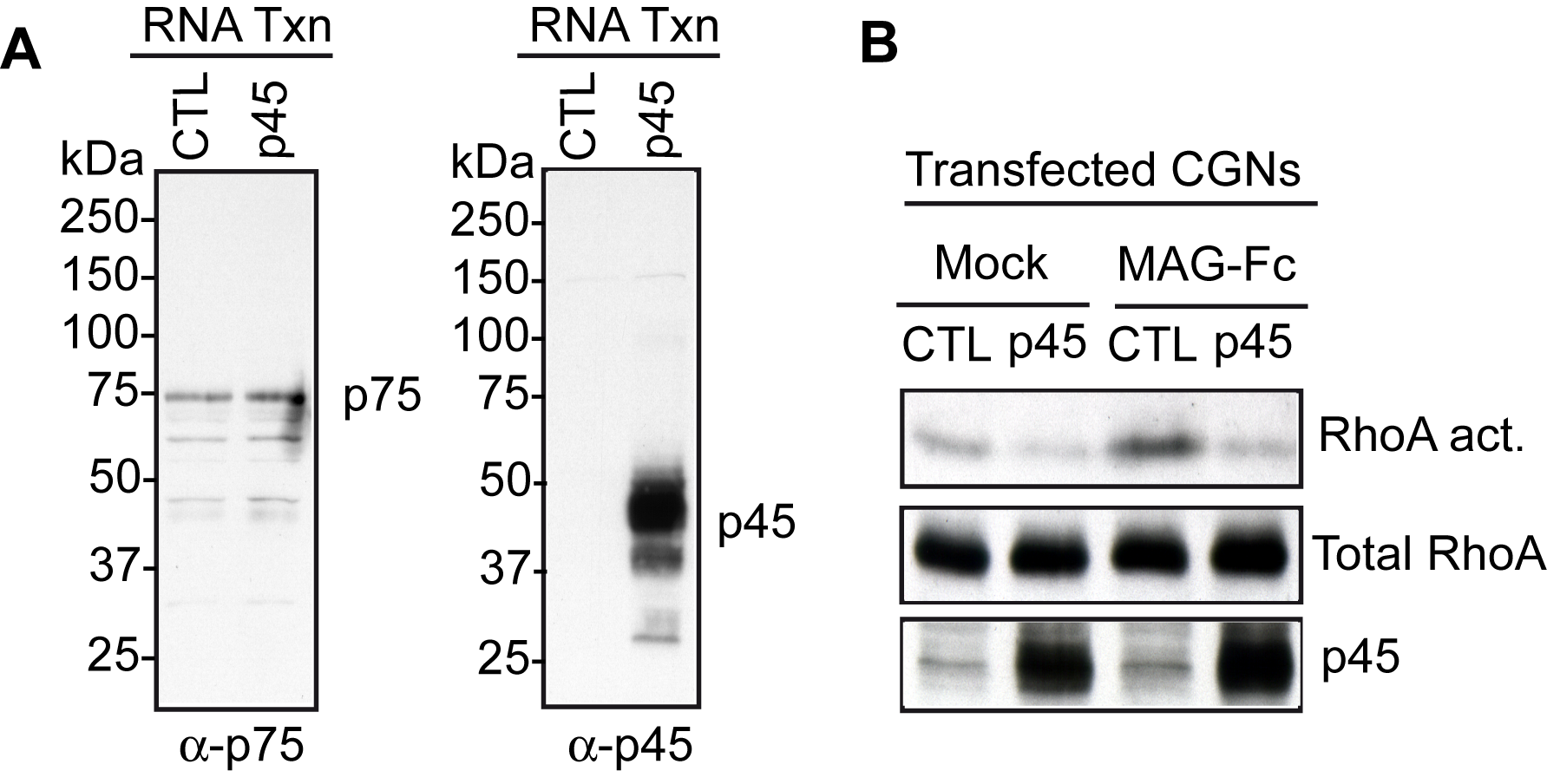

Supplement: Figure S3 — Overexpression of p45 inhibits MAG-Fc–induced RhoA activation. (A) Increased p45 protein levels in P5–P7 CGNs following transfection of p45 RNA. (B) Following transfection with the p45 RNA, GCNs were treated with MAG-Fc and subjected to a RhoA activity assay. Overexpression of p45 inhibited MAG-Fc–induced RhoA activation. (TIF) [file pbio.1001918.s003.tif]

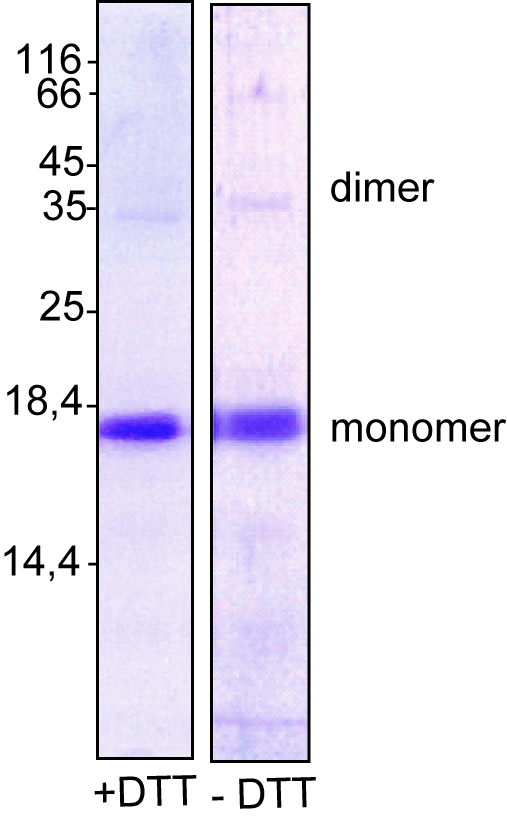

Supplement: Figure S4 — Iodoacetamide purification of p75-ICD. SDS-PAGE in reducing and nonreducing conditions of p75-ICD purified from E. coli using iodoacetamide, a blocking agent of free cysteines, in the lysis buffer. The absence of dimerized p75-ICD in these conditions suggests that p75-ICD dimerization is produced during the purification as a result of oxidation of free cysteines. (TIF) [file pbio.1001918.s004.tif]

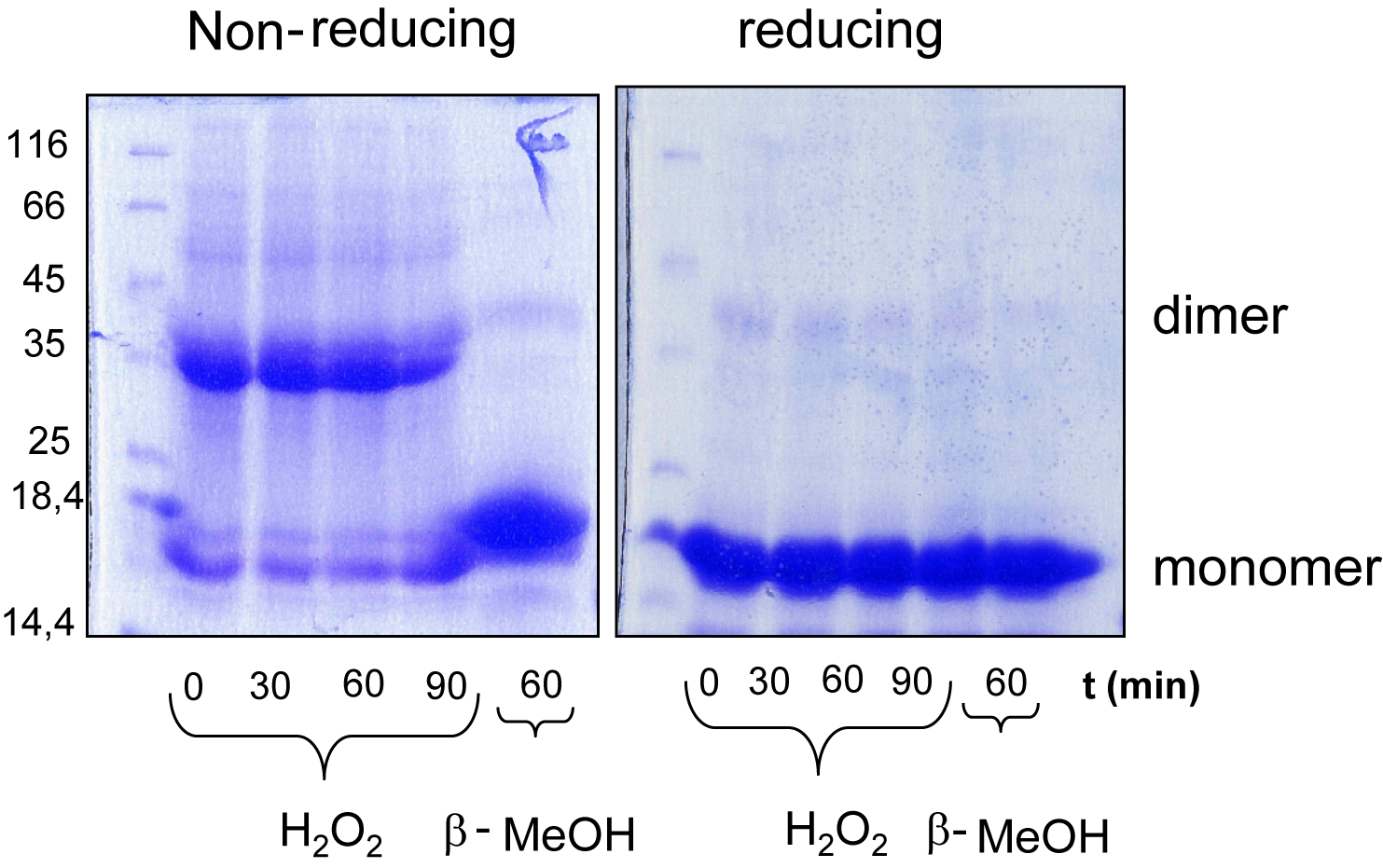

Supplement: Figure S5 — p75-ICD covalent disulfide dimmer formation in the presence of hydrogen perxiode. Nonreducing and reducing SDS-PAGE of purified p75-ICD from E. coli was incubated with hydrogen peroxide (10 mM) during the indicated time points. Note that p75-ICD purified from E. coli (without DTT, t = 0 min) has already some amount of disulfide dimer. (TIF) [file pbio.1001918.s005.tif]

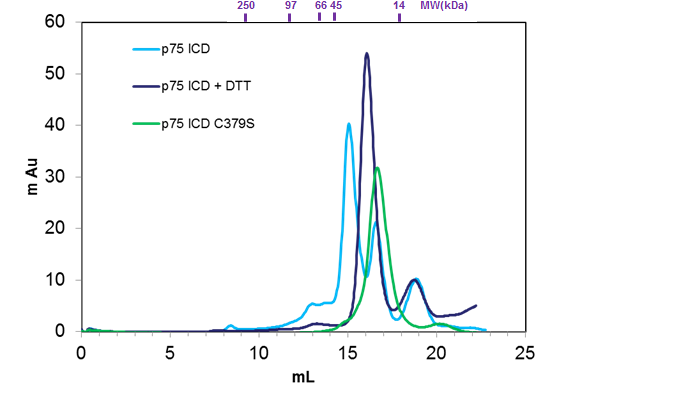

Supplement: Figure S6 — p75-Cys379 is responsible for disulfide dimerization of p75-ICD. Gel filtration profile of purified p75-ICD WT in reducing (blue) and nonreducing conditions (dark blue) and of purified p75-C379S (green). The elution of p75-C379S is indicative of a monomeric p75-ICD. (TIF) [file pbio.1001918.s006.tif]

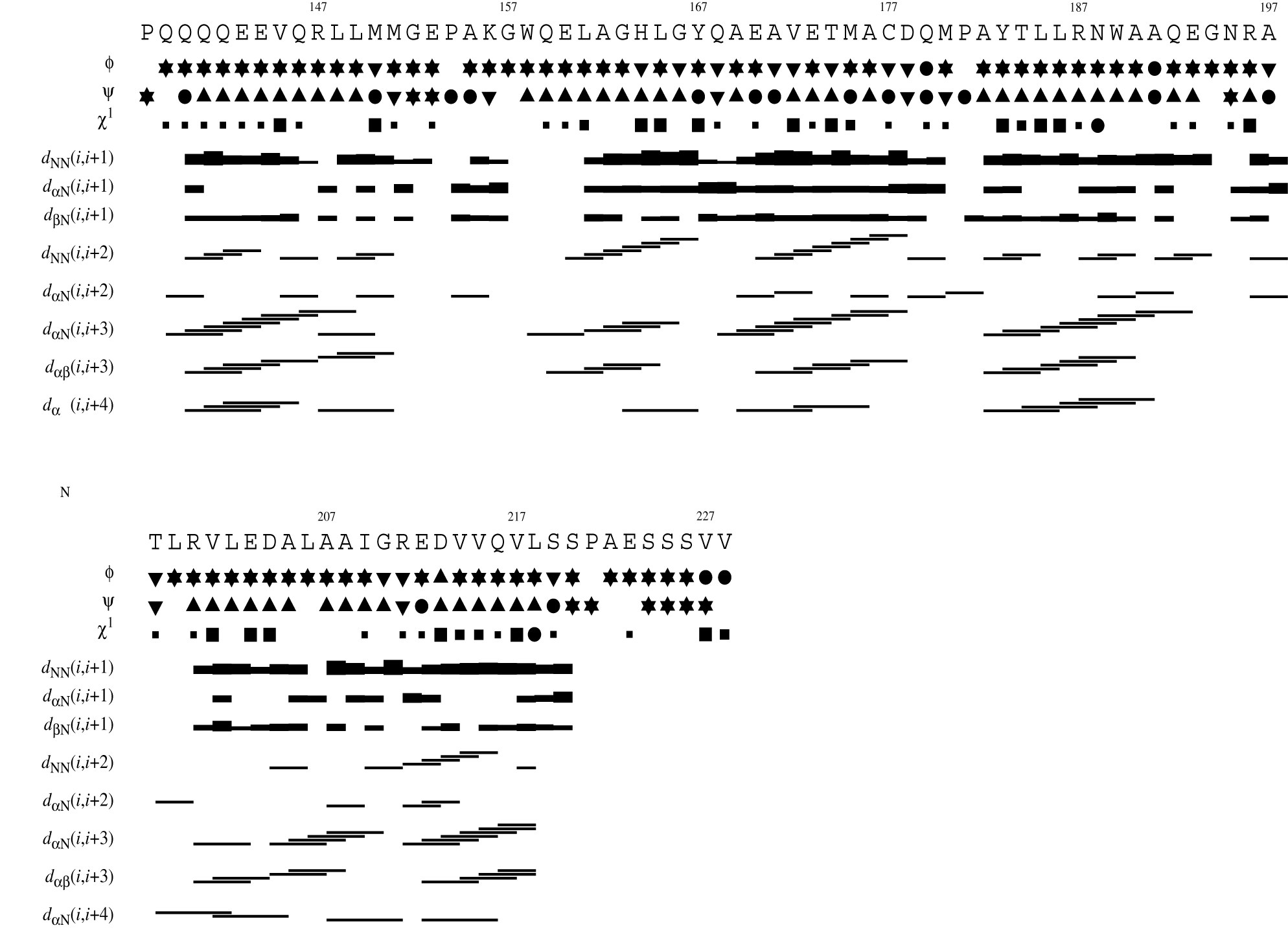

Supplement: Figure S7 — Summary of NOEs. Observed NOEs are summarized for p45ICD. Sequential NOEs are indicated by thick horizontal bars. The thickness of the bar is proportional to the magnitude of the NOE intensity. Thin horizontal bars indicate long-distance NOEs. (TIF) [file pbio.1001918.s007.tif]

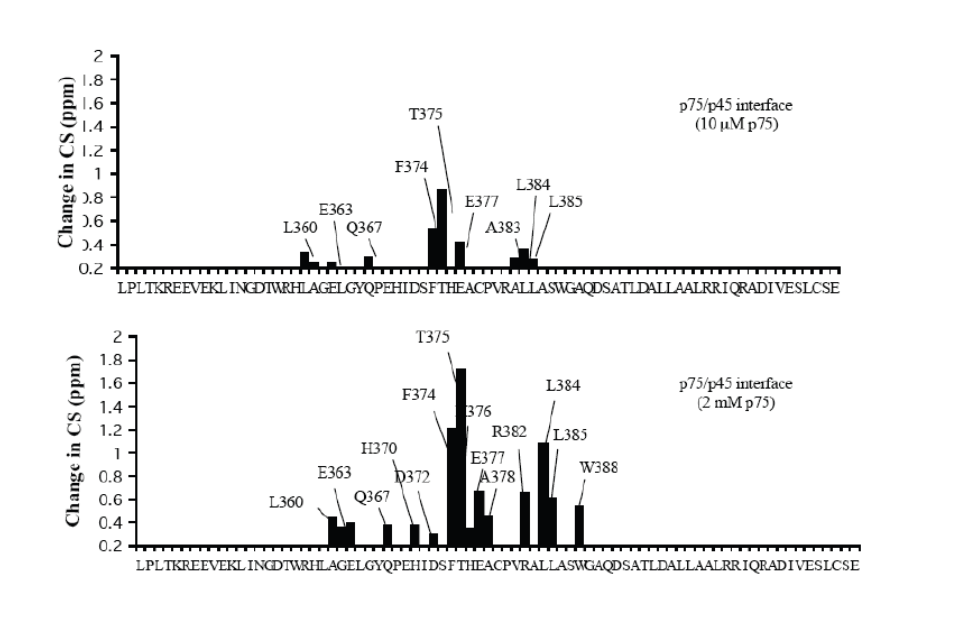

Supplement: Figure S8 — Insights into p45ICD-p75ICD heterodimer formation. p45ICD-dependent chemical shift changes versus the amino acid sequence observed in stable isotope-labeled p75ICD at (B) 10 µM and (C) 2 mM p75ICD concentration. The bar plot represents the normalized change of the chemical shifts of p75ICD in the absence and presence of p45ICD in the [15N,1H]-TROSY spectrum using the equation N = 25[Δ(δ(1H))2 + Δ(δ(15N))2]0.5, where δ(1H) and δ(15N) are the chemical shifts in part per million (ppm) along the ω2(1H) and ω2(15N) dimensions, respectively. Perturbations larger than 0.2 ppm are labeled. (TIF) [file pbio.1001918.s008.tif]

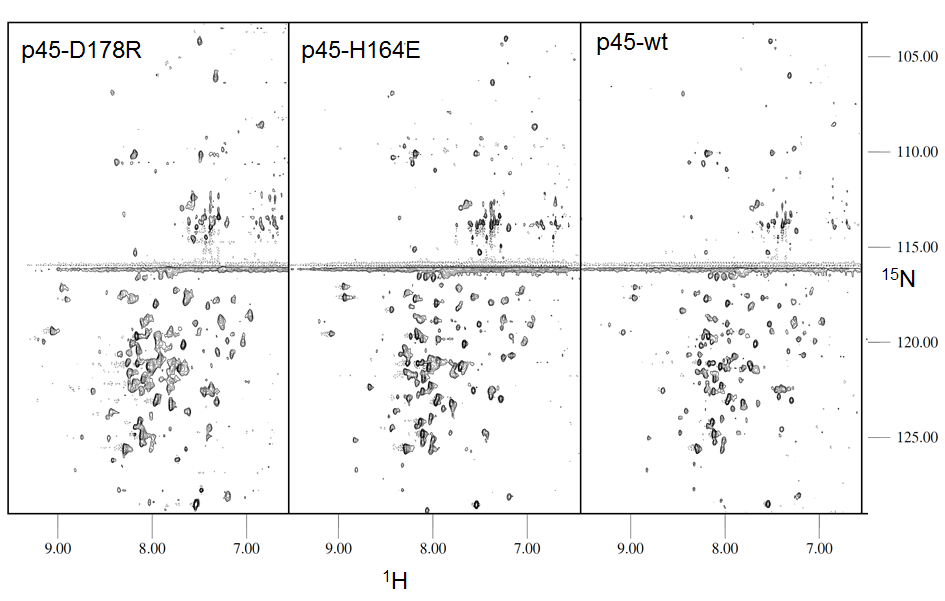

Supplement: Figure S9 — 2D-NMR of selected 15N-labelled p45-ICD mutants expressed in E. coli. p45 DD mutants were expressed and purified as 15N-labelled proteins and analyzed by NMR spectra, indicating that all p45 mutants are correctly folded like p45-WT. (TIF) [file pbio.1001918.s009.tif]

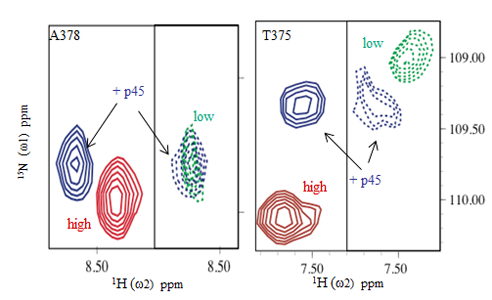

Supplement: Figure S10 — Insights into p45–p75 heterodimer formation from NMR. (A) NMR analysis of p45-DD and p75-DD interaction suggests a heterodimer formation. A378, red colored lines represent the cross-peak of A378 in the [15N,1H] TROSY spectrum at a high concentration of p75ICD (homodimer), and the corresponding cross-peak at a low concentration (monomer) of p75ICD is represented by green dashed lines, respectively. Upon p45ICD addition to a highly concentrated p75ICD sample, the cross-peak of A378 colored as blue lines shifted to the position of the monomer. In contrast, upon p45ICD addition to the sample with low p75ICD concentration, the cross-peak of A378—represented as blue dashed lines—did not shift. These findings indicate that A378 is not part of the p45–p75 interface and that p45 breaks the p75 homodimer. T375, the cross-peaks of T375 in the [15N,1H]-TROSY spectra at high and low p75ICD concentrations and in the presence and absence of p45ICD are displayed with the same color code as for A378. The addition of p45ICD at high p75ICD concentrations results in a shift of the cross-peak of T375 (red cross-peak to blue cross-peak). The cross-peak of T375 at low p75ICD concentrations is also shifted upon addition of p45ICD (cross-peak represented by green dashed lines to cross-peak represented with blue dashed lines). Because the position of the cross-peak of T375 in the presence of p45 is independent of the p75ICD concentration, T375 appears to be part of the p45–p75 interface as well as part of the p75–p75 interface. (TIF) [file pbio.1001918.s010.tif]

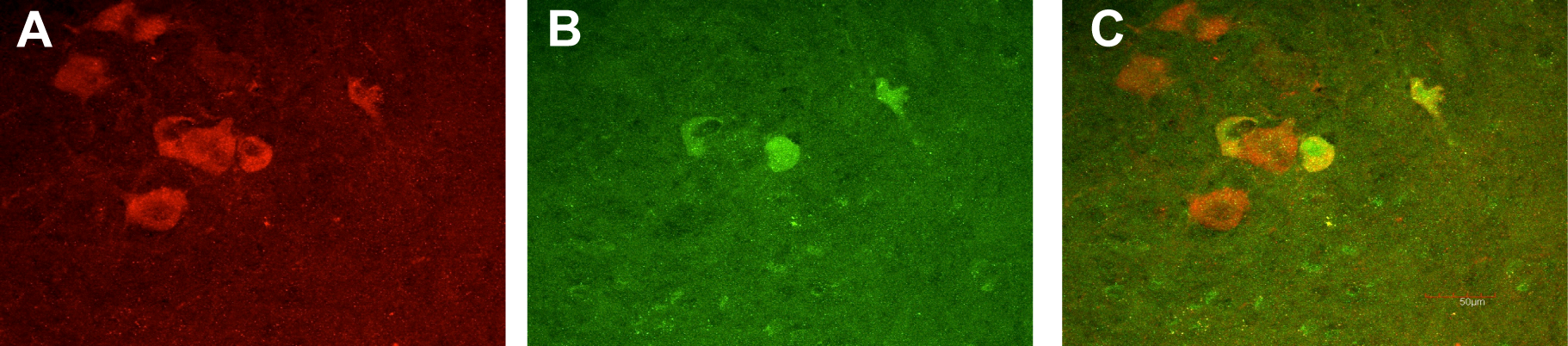

Supplement: Figure S11 — Co-localization of p75 and p45 expression in lumbar spinal motorneurons after sciatic nerve crush. Confocal images of immunofluorescence staining of spinal cord sections from mice with the sciatic nerve crush. (A) p75 staining, (B) p45 staining, and (C) merge of (A) and (B). Some p75-expressing motor neurons also express p45. (TIF) [file pbio.1001918.s011.tif]
