# Supplementary material for: Heterodimerization of p45–p75 Modulates p75 Signaling: Structural Basis and Mechanism of Action
Source: PLoS Biol. 2014 Aug 5;12(8):e1001918. doi: 10.1371/journal.pbio.1001918 (PMC4122344; doi:10.1371/journal.pbio.1001918)
Supplement: Table S2 — NMR and structural statistics of the 3D structure of p45-DD. (DOCX) [file pbio.1001918.s013.docx]

**Table S2: NMR and structural statistics of the 3D structure of p45-DD.**

| Constraints |
| --- |
| No. of NOE upper distance limits 1053 |
| No. of dihedral angle constraints 266 |
| Residual target function (Å^2^) 0.51 ± 0.13 |
| No. of residual NOE violations > 0.2 Å 4 |
| Maximum (Å) 4.5 ± 0.6 |
|  |
| Energies (kJ/mol) |
| Total -1167.53 ± 49.69 |
| Van der Waals -174.46 ± 17.70 |
| Electrostatic -1159.49 ± 52.00 |
|  |
| Atomic pairwise rmsd (Å) |
| Backbone atoms 1.06 |
| Residues 140-149, 158-165, 169-175, |
| 181-188, 201-207 and 212-218 0.84 |
| Heavy atoms 1.39 |
|  |
| Structural analysis (residues 138-220) |
| Residues in disallowed regions (%) 2.0 |
| Residues in generously allowed regions (%) 3.2 |
| Residues in allowed regions (%) 20.6 |
| Residues in most favorable regions (%) 74.2 |
