# Supplementary material for: Heterodimerization of p45–p75 Modulates p75 Signaling: Structural Basis and Mechanism of Action
Source: PLoS Biol. 2014 Aug 5;12(8):e1001918. doi: 10.1371/journal.pbio.1001918 (PMC4122344; doi:10.1371/journal.pbio.1001918)
Supplement: Table S3 — Structural homology search with DALI Server showing the top 10 matches. (DOCX) [file pbio.1001918.s014.docx]

**Table S3**: Structural Homology Search with DALI Server showing the top 10 matches.

| Name | Z score | # Residues aligned | RMSD (Å) |
| --- | --- | --- | --- |
| p75 DD | 7.6 | 77 | 2.7 |
| IRAK4 DD | 5.4 | 75 | 2.8 |
| Tho1 DD | 5.2 | 76 | 2.9 |
| Pelle DD | 5.2 | 76 | 3.3 |
| RAIDD DD | 5.1 | 74 | 2.9 |
| NF-kB p100 DD | 5.0 | 74 | 3.4 |
| FADD DED | 4.6 | 73 | 3.7 |
| Caspase9 CARD | 4.5 | 74 | 4.1 |
| TNFR1 DD | 4.3 | 76 | 3.4 |
